# Considerations in the identification of functional RNA structural elements in genomic alignments

**DOI:** 10.1186/1471-2105-8-33

**Published:** 2007-01-30

**Authors:** Tomas Babak, Benjamin J Blencowe, Timothy R Hughes

**Affiliations:** 1 Banting and Best Department of Medical Research, Donnelly Centre for Cellular and Biomolecular Research, 160 College St, Toronto, ON M5S 3E1 Canada; 2 Department of Medical Genetics and Microbiology, 10 King's College Circle, Toronto, ON M1R 4F9 Canada

## Abstract

**Background:**

Accurate identification of novel, functional noncoding (nc) RNA features in genome sequence has proven more difficult than for exons. Current algorithms identify and score potential RNA secondary structures on the basis of thermodynamic stability, conservation, and/or covariance in sequence alignments. Neither the algorithms nor the information gained from the individual inputs have been independently assessed. Furthermore, due to issues in modelling background signal, it has been difficult to gauge the precision of these algorithms on a genomic scale, in which even a seemingly small false-positive rate can result in a vast excess of false discoveries.

**Results:**

We developed a shuffling algorithm, shuffle-pair.pl, that simultaneously preserves dinucleotide frequency, gaps, and local conservation in pairwise sequence alignments. We used shuffle-pair.pl to assess precision and recall of six ncRNA search tools (MSARI, QRNA, ddbRNA, RNAz, Evofold, and several variants of simple thermodynamic stability on a test set of 3046 alignments of known ncRNAs. Relative to mononucleotide shuffling, preservation of dinucleotide content in shuffling the alignments resulted in a drastic increase in estimated false-positive detection rates for ncRNA elements, precluding evaluation of higher order alignments, which cannot not be adequately shuffled maintaining both dinucleotides and alignment structure. On pairwise alignments, none of the covariance-based tools performed markedly better than thermodynamic scoring alone. Although the high false-positive rates call into question the veracity of any individual predicted secondary structural element in our analysis, we nevertheless identified intriguing global trends in human genome alignments. The distribution of ncRNA prediction scores in 75-base windows overlapping UTRs, introns, and intergenic regions analyzed using both thermodynamic stability and EvoFold (which has no thermodynamic component) was significantly higher for real than shuffled sequence, while the distribution for coding sequences was lower than that of corresponding shuffles.

**Conclusion:**

Accurate prediction of novel RNA structural elements in genome sequence remains a difficult problem, and development of an appropriate negative-control strategy for multiple alignments is an important practical challenge. Nonetheless, the general trends we observed for the distributions of predicted ncRNAs across genomic features are biologically meaningful, supporting the presence of secondary structural elements in many 3' UTRs, and providing evidence for evolutionary selection against secondary structures in coding regions.

## Background

One of the major findings of genome sequencing has been that the primary sequence of roughly 5% of the human and mouse genomes is under purifying selection, indicating functionality [1]. However, less than 2% is accounted for by mRNA exons. The remaining 3% presumably encompasses cis-regulatory sequence, signals for transcriptional initiation, termination, RNA processing, chromosomal features such as replication origins, and genes encoding ncRNAs such as tRNA, snoRNA, miRNA, and others.

Accurate computational identification of novel ncRNA genes and mRNA structural elements (as opposed to known classes) in genome sequence has proven to be more difficult than identification of exons, due generally to a limited and highly variable sequence signature [2]. In bacteria, which have compact genomes, searching for transcription initiation signals [3], primary sequence conservation [4], and base composition [5] have been fruitful approaches to *de novo *ncRNA discovery; however, these features alone are unlikely to be sufficiently specific in large eukaryotic genomes.

Most, albeit not all, functional ncRNA features possess some degree of secondary structure, either as part of the precursor or the functional RNA itself. Following the assumption that structural RNA sequences should be more thermodynamically stable than random permutations of the same base composition, thermodynamic stability (Δ*G*) is an additional feature than can be incorporated into genomic searches for new ncRNAs. Major classes of structural RNAs have lower Δ*G *than corresponding shuffled sequences. It has been debated whether Δ*G *is a sufficiently accurate discriminant when only a single (i.e. unaligned) sequence is analyzed [6]; however, Δ*G *has been proposed to be comparable or superior to more sophisticated algorithms (see below) when applied independently to segments of a pairwise alignment [7]. What is clear is that disruption of dinucleotides in the random permutation dramatically affects the perceived precision of predictions [7,8], presumably because dinucleotide contributions are a key determinant of stability of an RNA fold.

Covariance (i.e. scoring for apparent compensatory mutations in secondary structures in sequence alignments) is also now a widely-accepted approach to ncRNA discovery. A variety of recently-described ncRNA search algorithms (QRNA [9], RNAz [10], ddbRNA [11], MSARI [12], and Evofold [13]) score for covariance to discriminate structural RNA elements (Table 1). Success of covariance requires that sequences be sufficiently conserved to achieve a correct alignment, yet contain some nucleotide changes in order to assess compensatory mutations. An advantage of methods that do not utilize covariance is that they can identify structures common to sequences without high sequence similarity [14] and apparently even sequences that fail to align at the primary sequence level [15]. However, even considering both covariance and thermodynamic stability, some classes of ncRNAs appear to be more difficult to detect than others [16].

**Table 1:** Overview of ncRNA search tools assessed in this study

**Tool**	**Description**	**URL**	**Ref**
MSARI	Developed for use with 10–15 multiple alignments, looks for compensatory mutations near bases predicted to pair in secondary structure. Uses RNAfold [33] to predict which bases pair and analyzes neighbouring nucleotide pairs in a 7 nt window for presence of compensatory mutations.		[12]
RNAz	Uses two variables to assess ncRNA potential in two or more sequence alignments: a *z*-score and an *SCI *score. The *z*-score is a measure of the thermodynamic stability of the reference sequence relative to shuffled variants of the same sequence. RNAz samples the *z*-scores using machine learning from pre-computed *z*-scores covering a range of sequence lengths and sequence compositions, rather than re-computing *z*-scores for each sequence. The *SCI *(structure conservation index) is an indirect measure of structural similarity among the individual sequences in the alignment based on their individual folding energies compared to the folding energy of their consensus structure. This term also incorporates a covariance factor, rewarding compensatory mutations. RNAz uses machine learning to classify alignments as ncRNAs on a combination of their *z*- and *SCI *scores. Scores reflect how far the alignment is from the ncRNA line of separation in an *SCI*-*z *plane.		[10]
ddbRNA	ddbRNA scans for compensatory mutations in conserved stem loops found within two or more sequence alignments. It counts the number of compensatory mutations in all possible hairpins of the alignment and compares that count to a distribution of counts from shuffled variants of the alignment. The score output is the number of standard deviations above the mean count of the shuffled variants (negative scores indicate more compensatory mutations in the shuffled variants).	Contact authors.	[11]
QRNA	QRNA is a probabilistic algorithm that predicts pairwise sequence alignments as belonging to one of three classes: coding (COD), noncoding (RNA), or other (OTH). The RNA model incorporates two components: a stochastic-context free grammar (SCFG) to estimate a distribution of probabilities over potential structures and a pair-Hidden Markov Model to predict the probability that the structures were evolutionarily selected on the basis of a compensatory substitution pattern in the alignment. The logoddspostRNA score reflects these two components and is used as the primary discriminant score in this study.		[9]
Evofold	Evofold computes the probability that the observed sequence alignment was generated under selection for a functional RNA (structural) versus evolutionary divergence of non-structural sequence. It uses the traditional SCFG algorithm CYK without explicitly defining emission probabilities over observed aligned bases, but rather uses an evolutionary model (Felsenstein) to compute the probability of the column given a phylogenetic tree. The functional RNA (fRNA) model comprises both a structural and non-structural components. The structural component computes the probability that two columns pair (i.e. occur in stems) whereas the non-structural component computes the probability of observing single-nucleotide columns.		[13]
zMFOLD	This is in an iterative script that utilizes hybrid-ss-min (MFOLD variant with updated handling of partition function calculations) [34] and shuffle-pair.pl to generate *z*-scores that are indicative of selection for thermodynamic stability over the expected stability of a random sequence with an identical dinucleotide composition. The *z*-score is the number of standard deviations that the actual stability is below the mean stability of 100 shuffles (using shuffle-pair.pl). Higher *z*-scores indicate apparent selection for structural stability. The thermodynamic stability is calculated only for the reference strand, while the alignment constrains the shuffling.	hybrid/OligoArrayAux.php tools/zMFOLD.pl (perl script that calls hybrid-ss-min and shuffle-pair.pl) Additional file 4	[34]
zRNAfold	Same as zMFOLD except RNAfold [35] is used to predict thermodynamic stabilities.		[35]
zRNAfold (Dual)	*z*-scores are computed with zRNAfold for both strands in the alignment and added for a final *z*-score.		[35]
Alifoldz	Predicts selection for structure by comparing the minimum free energy of a consensus structure to shuffles of the alignment. Scores are multiplied by -1 to scale with other algorithms where higher scores indicate structure.		[18]

To our knowledge, most ncRNA search tools have not been assessed or compared systematically by an independent laboratory. Moreover, previous studies using these tools for genomic scanning have acknowledged that the false-positive rate is high and cannot be determined accurately [13,16]. Here, with the goal of independently evaluating these tools for *de novo *discovery of ncRNA elements in a genomic context, we have compiled an extensive sequence and alignment data set, and developed a new shuffling algorithm for pairwise alignments (shuffle-pair.pl) that simultaneously preserves key features of the alignment. We used these sequences, as well as real and shuffled genomic sequence, as input for a panel of published tools for discovery of novel ncRNAs. We also evaluated whether different schemes utilizing only thermodynamic stability could discriminate real from shuffled ncRNAs, and examined the output of a subset of tools on a genomic scale.

## Results

### A test set for comparing ncRNA-finding tools

We tested the ncRNA search tools (Table 1) on a test set derived by extracting 3046 genomic alignments corresponding to known eukaryotic ncRNAs and mRNA structural elements (see Methods for details). The positive test set is available at [17] and in Additional files 7 and 8, and consists of 1303 miRNA, 213 tRNA, 225 Box H/ACA snoRNA, 581 Box C/D snoRNA, 227 snRNA, 182 UTR regulatory element alignments, in addition to 94 other structural ncRNA alignments (such as 7SK, RNAse P, and RNAse MRP) and 220 non-structural RNA alignments (such as H19, Hoxa-11 antisense transcript, XIST) for a total of 282,221 bases. We generated the alignments by mapping all available ncRNA sequences from human, mouse, *C. elegans*, *D. melanogaster*, and *S. cerevisiae*, to available UCSC pairwise genomic alignments (see Methods).

**Figure 1:**
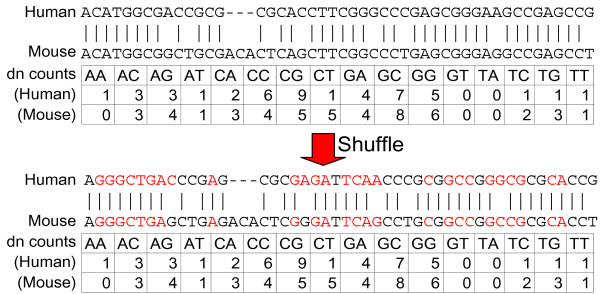
**Shuffling pairwise sequence alignments**. Example of human-mouse pairwise alignments before and after shuffling with shuffle-pair.pl, illustrating that dinucleotide frequencies, sequence composition, gaps, and local conservation are maintained during shuffling.

To obtain negative controls, we created 100 sequence shuffles of each sequence alignment using a new shuffling algorithm, shuffle-pair.pl (Figure 1, see Methods for details and Additional file 3 for the program itself), to simulate the problem of finding *bona fide *structural elements against a much larger background that resembles true genomic alignments in as many features as possible. Briefly, the shuffle-pair.pl algorithm randomizes the columns of the alignment while preserving conservation, gaps, and dinucleotides in both sequences, all of which may contribute to scoring obtained from at least a subset of algorithms tested [6,8,13]. It shuffles by swapping the middle bases between trinucleotides that are identical at positions 1 and 3 as described [8] with the additional constraints of swapping in aligned trinucleotides and maintaining conservation and gaps of the alignment. The algorithm finds all swappable positions that preserve the criteria above and then randomly selects two bases to swap. It keeps track of positions that have been swapped and continues shuffling until it runs out of positions to shuffle. A detailed description of the algorithm and pseudocode is found in the Methods section. On average, miRNA alignments shuffled 57% (i.e. 57% of nucleotides changed identity after shuffling), tRNA alignments – 61%, snRNA alignments – 62%, snoRNA alignments – 46%, and regulatory elements – 43%; overall the 3046 alignments shuffled on average 53%. We did not observe a correlation between the degree of shuffling and the scores output by tools (data not shown).

We observed that the degree of shuffling possible depends on sequence length and conservation: increasing length and conservation increased the shufflability of the alignments, presumably due to more shuffling opportunities resulting from more bases and less constraint imposed by preserving local conservation patterns. Conversely, increasing the number of aligned sequences greatly diminishes shuffling potential due the constraint of preserving dinucleotides in all aligned sequences. Consequently, here we consider only findings pertaining to pairwise alignments. We acknowledge that we are not taking full advantage of MSARI, ddbRNA, Evofold, and RNAz, since these tools were designed to handle multiple sequence alignments. Nonetheless, we reasoned that it would be of value to examine the outputs of these tools under a situation that controls for all variables believed to be relevant.

We ran all of the tools with default settings. All but one of the tools ran without giving error messages. RNAz produces error messages with sequences shorter than 50 and longer than 400 nt and for alignments with very low sequence identity, but still produces scores that we retained for the analysis since (a) only a small proportion of the test set (5.2% is <50 nt; 0.005% > 400 nt; 5.5% < 50% identical) falls into this category, and (b) and challenges imposed in scoring these alignments apply to all of the tools. MSARI output was excluded from subsequent evaluation due to its extremely poor performance on alignments of 2 sequences (it was designed for alignments of 10–15 sequences [12] which cannot be shuffled to any significant degree using our algorithm).

### Overview of results on test set

For each of the tools, we scored precision (TP/(TP+FP)) and recall (TP/(TP+FN)) (identical to sensitivity) across its range of output scores. In comparison to area under the ROC curve, and specificity (TN/(TN+FP)), we view precision and recall as most relevant to real-world genomic screening: precision estimates the false-positive rate one would face if experimentally verifying predicted ncRNAs, and recall estimates the proportion of all ncRNAs that could be found.

**Figure 2:**
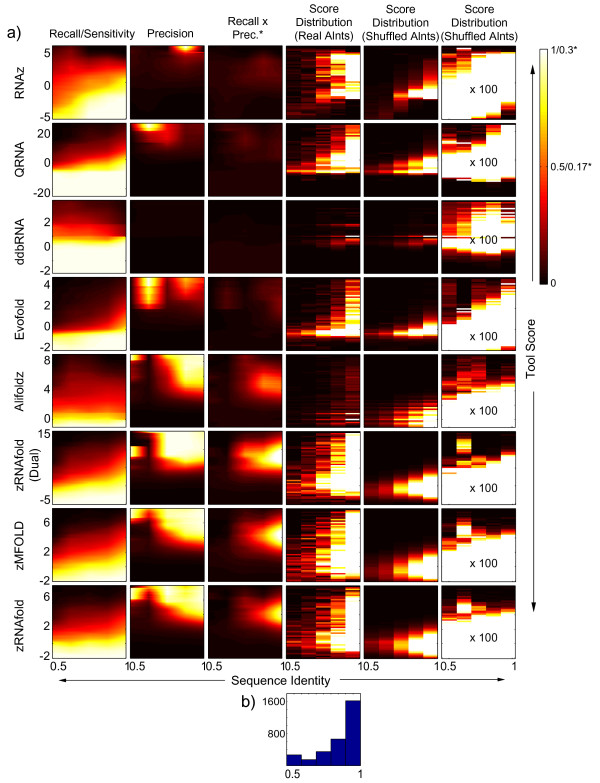
**Dependency of precision and recall on score threshold and level of conservation**. **a) **Results from eight ncRNA search tools run on our test set of pairwise UCSC alignments of ncRNAs compiled from human, mouse, worm, fly, and yeast ncRNA databases (n = 3046). We calculated recall and precision for 100 output score thresholds distributed linearly over the range of output scores, and for five ranges of sequence identity (50–100%). (Only 5.5% of the alignments fall below 50% sequence identity.) Precision and recall were calculated using a shuffled test set with of 100 shuffles for each real ncRNA alignment. The middle column depicts the product of precision and recall (*intensity scaled such that white = 0.3). The two columns on the right show the actual distribution of scores attained for real alignments (third from right) and shuffled alignments (second from right) normalized to the count of the most recurrent score. The far right column shows the shuffled score distribution normalized to the real score counts (100× brighter) to reveal presence of high-scoring shuffles (i.e. false positives that limit precision). **b) **Distribution of the sequence identity of the pairwise alignments. (Similar figures for ncRNAs in eight different classes are shown in Supplementary Figures 1-8 [Additional file 1]).

Table 2 summarizes the results for each tool, with a breakdown of the precision over eight categories of ncRNAs (snRNA, Box H/ACA snoRNA, Box C/D snoRNA, tRNA, regulatory element, miRNA, other "structural" and "non-structural"), at three thresholds corresponding to overall recall of 15%, 50%, and 85%. For most tools and most RNA classes, we observed that results appear to correlate with degree of conservation; this is shown in Figure 2 and Supplementary Figures 1 through 8 (in Additional file 1) for individual ncRNA classes. In this analysis we defined % sequence identity as the average of nucleotide conservation calculated for each column of the alignment; i.e. the average of a series of ones and zeros where a match contributed one, and mismatches and gaps contributed zero. In these figures we also show the maximum product of precision and recall to provide a single summary score for how well a tool separates the real alignments from their shuffled variants at different scores and conservation levels. For example, an ideal tool would have a score threshold at which it attains 100% precision and 100% recall at all conservation levels. We also show the actual score distributions as well as histograms of the conservation levels of these classes in the test set.

**Table 2:** Details of results on test set, tabulated at 15%, 50%, and 85% overall recall

		**15% overall recall**			**50% overall recall**			**85% overall recall**		
	
	Sample size	Proportion positive	Number positive	Precision	Proportion positive	Number positive	Precision	Proportion positive	Number positive	Precision
**RNAz**										
miRNA	1303	0.208	271	0.158	0.743	968	0.070	0.992	1293	0.017
tRNA	213	0.099	21	0.103	0.723	154	0.078	0.981	209	0.016
snRNA	227	0.141	32	0.375	0.379	86	0.071	0.846	192	0.014
Box H/ACA snoRNA	222	0.252	56	0.356	0.541	120	0.094	0.887	197	0.018
Box C/D snoRNA	573	0.012	7	0.029	0.092	53	0.029	0.625	358	0.013
Regulatory	181	0.293	53	0.047	0.431	78	0.045	0.823	149	0.019
Other	94	0.117	11	0.538	0.362	34	0.042	0.777	73	0.011
Non-structural	215	0.023	5	0.049	0.102	22	0.029	0.484	104	0.012
All	3029	0.151	456	0.123	0.500	1515	0.064	0.850	2576	0.016
Negatives – shuffle-pair	300399	0.011	3216	NA	0.071	21315	NA	0.585	175584	NA
Negatives – shuffle-aln	299784	0.001	407	NA	0.023	7044	NA	0.595	178278	NA
**QRNA**										
miRNA	1303	0.293	382	0.065	0.802	1045	0.069	0.972	1267	0.029
tRNA	213	0.103	22	0.030	0.803	171	0.039	0.911	194	0.023
snRNA	227	0.070	16	0.009	0.278	63	0.022	0.797	181	0.014
Box H/ACA snoRNA	222	0.023	5	0.023	0.194	43	0.020	0.829	184	0.017
Box C/D snoRNA	573	0.012	7	0.004	0.143	82	0.027	0.705	404	0.025
Regulatory	181	0.039	7	0.020	0.337	61	0.038	0.878	159	0.028
Other	94	0.064	6	0.011	0.255	24	0.019	0.798	75	0.016
Non-structural	215	0.047	10	0.004	0.126	27	0.008	0.516	111	0.018
All	3029	0.150	455	0.037	0.501	1516	0.048	0.850	2576	0.025
Negatives – shuffle-pair	302377	0.035	10452	NA	0.117	35389	NA	0.493	149137	NA
Negatives – shuffle-aln	299784	0.009	2610	NA	0.038	11463	NA	0.626	187643	NA
**ddbRNA**										
miRNA	1303	0.195	254	0.073	0.800	1042	0.038	0.975	1270	0.011
tRNA	213	0.136	29	0.023	0.901	192	0.032	0.981	209	0.009
snRNA	227	0.141	32	0.049	0.674	153	0.021	0.815	185	0.007
Box H/ACA snoRNA	222	0.243	54	0.081	0.644	143	0.020	0.838	186	0.010
Box C/D snoRNA	573	0.044	25	0.018	0.356	204	0.013	0.660	378	0.006
Regulatory	181	0.210	38	0.034	0.641	116	0.022	0.796	144	0.008
Other	94	0.128	12	0.032	0.649	61	0.016	0.787	74	0.007
Non-structural	215	0.051	11	0.009	0.363	78	0.009	0.628	135	0.007
All	3029	0.150	455	0.046	0.657	1990	0.025	0.852	2582	0.009
Negatives – shuffle-pair	303277	0.031	9251	NA	0.607	183998	NA	0.921	279240	NA
Negatives – shuffle-aln	299784	0.012	3546	NA	0.363	108707	NA	0.620	185895	NA
**Evofold**										
miRNA	1303	0.324	422	0.243	0.836	1089	0.044	0.968	1261	0.018
tRNA	213	0.009	2	0.000	0.601	128	0.026	0.953	203	0.014
snRNA	227	0.101	23	0.409	0.370	84	0.017	0.811	184	0.013
Box H/ACA snoRNA	222	0.009	2	0.333	0.297	66	0.074	0.671	149	0.032
Box C/D snoRNA	573	0.002	1	0.500	0.082	47	0.018	0.757	434	0.011
Regulatory	181	0.006	1	0.000	0.210	38	0.040	0.939	170	0.012
Other	94	0.000	0	0.000	0.213	20	0.027	0.543	51	0.018
Non-structural	215	0.019	4	0.056	0.195	42	0.027	0.591	127	0.016
All	3029	0.150	455	0.240	0.500	1515	0.038	0.852	2580	0.017
Negatives – shuffle-pair	304600	0.013	3809	NA	0.199	60544	NA	0.768	234058	NA
Negatives – shuffle-aln	299784	0.010	3115	NA	0.151	45175	NA	0.773	231828	NA
**Alifoldz**										
miRNA	1290	0.307	396	0.916	0.650	838	0.055	0.940	1213	0.016
tRNA	201	0.010	2	0.091	0.731	147	0.046	0.995	200	0.013
snRNA	226	0.009	2	0.667	0.416	94	0.048	0.925	209	0.013
Box H/ACA snoRNA	219	0.018	4	1.000	0.493	108	0.043	0.932	204	0.018
Box C/D snoRNA	543	0.009	5	0.667	0.210	114	0.026	0.727	395	0.010
Regulatory	175	0.154	27	0.043	0.537	94	0.034	0.943	165	0.018
Other	92	0.033	3	1.000	0.207	19	0.058	0.848	78	0.013
Non-structural	198	0.025	5	0.833	0.298	59	0.020	0.717	142	0.011
All	2945	0.151	444	0.369	0.500	1473	0.045	0.885	2607	0.014
Negatives – shuffle-pair	290247	0.003	778	NA	0.145	42219	NA	0.558	161948	NA
Negatives – shuffle-aln	299784	0.000	74	NA	0.499	149651	NA	0.761	228278	NA
**zRNAfold (Dual)**										
miRNA	1303	0.347	452	0.987	0.927	1208	0.399	0.992	1293	0.024
tRNA	213	0.000	0	0.000	0.221	47	0.065	0.981	209	0.020
snRNA	227	0.004	1	0.000	0.291	66	0.139	0.802	182	0.018
Box H/ACA snoRNA	222	0.000	0	0.000	0.311	69	0.286	0.883	196	0.026
Box C/D snoRNA	573	0.000	0	0.000	0.045	26	0.029	0.642	368	0.017
Regulatory	181	0.006	1	0.000	0.354	64	0.056	0.895	162	0.019
Other	94	0.000	0	0.000	0.255	24	0.112	0.755	71	0.015
Non-structural	215	0.005	1	1.000	0.051	11	0.139	0.433	93	0.015
All	3029	0.150	455	0.746	0.500	1515	0.249	0.850	2575	0.021
Negatives – shuffle-pair	301496	0.000	150	NA	0.019	5859	NA	0.454	136915	NA
Negatives – shuffle-aln	299784	0.000	27	NA	0.015	4530	NA	0.421	126347	NA
**zRNAfold**										
miRNA	1303	0.345	450	0.968	0.912	1188	0.339	0.992	1292	0.022
tRNA	213	0.000	0	0.000	0.225	48	0.068	1.000	213	0.019
snRNA	227	0.009	2	0.000	0.273	62	0.148	0.811	184	0.017
Box H/ACA snoRNA	222	0.000	0	0.000	0.365	81	0.213	0.883	196	0.022
Box C/D snoRNA	573	0.002	1	0.500	0.075	43	0.031	0.620	355	0.014
Regulatory	181	0.000	0	0.000	0.331	60	0.051	0.845	153	0.018
Other	94	0.011	1	1.000	0.234	22	0.080	0.766	72	0.016
Non-structural	215	0.009	2	1.000	0.056	12	0.047	0.507	109	0.015
All	3029	0.151	456	0.806	0.501	1516	0.201	0.850	2575	0.019
Negatives – shuffle-pair	302578	0.000	143	NA	0.024	7292	NA	0.484	146426	NA
Negatives – shuffle-aln	299784	0.000	27	NA	0.020	5958	NA	0.484	145172	NA
**zMFOLD**										
miRNA	1303	0.342	446	0.943	0.906	1181	0.162	0.992	1293	0.023
tRNA	213	0.000	0	0.000	0.075	16	0.016	0.995	212	0.019
snRNA	227	0.000	0	0.000	0.242	55	0.059	0.802	182	0.015
Box H/ACA snoRNA	222	0.000	0	0.000	0.486	108	0.101	0.919	204	0.020
Box C/D snoRNA	573	0.002	1	0.167	0.072	41	0.014	0.593	340	0.012
Regulatory	181	0.022	4	0.017	0.365	66	0.034	0.812	147	0.016
Other	94	0.021	2	1.000	0.245	23	0.051	0.723	68	0.013
Non-structural	215	0.009	2	0.400	0.116	25	0.026	0.595	128	0.012
All	3029	0.150	455	0.675	0.500	1515	0.093	0.850	2575	0.018
Negatives – shuffle-pair	302950	0.001	241	NA	0.054	16498	NA	0.515	156071	NA
Negatives – shuffle-aln	299784	0.000	39	NA	0.042	12716	NA	0.522	156435	NA

Our results are consistent with expectation in several ways. First, taking an operating point at which each tool yields ~50% overall recall, the recall of different classes is ranked on average miRNA > tRNA > Regulatory > Box H/ACA snoRNA > snRNA > Other (Table 2), roughly as has been described by others (e.g. [16]). Algorithms utilizing only thermodynamic stability appear to have a relative advantage in detecting miRNAs, presumably due to the simple hairpin structure. At the same time, they have relative difficulty identifying tRNAs, perhaps due to the more complicated fold. Box C/D snoRNAs and non-structural elements were virtually indistinguishable from shuffles. We note that some of the tools may have been trained or their parameters optimized using a portion of our test set, and this may slightly bias outcome. EvoFold, for example, was trained on a large set of ncRNAs including many miRNAs [13], and it does perform well on miRNAs, even in our pairwise alignments; however, we confirmed that results for EvoFold are virtually identical if its training set RNAs are omitted [see Additional file 2], presumably because it does not learn any structural or sequence information (Table 1), and therefore results for the full data set are shown. Nonetheless, on the whole, the tools appear to detect different ncRNA classes in a roughly similar manner at the 50% recall operating point (Table 2). At lower overall recall (15%, Table 2) the results become less comparable due to a strong bias towards detection of miRNAs by the thermodynamics-only tools. At higher recall (85%), all tools suffer from low precision (discussed in more detail below).

Our results also verify the recent demonstration of Uzilov et al. [7] that, on pairwise alignments, Z-scores based on Δ*G *scores for both strands yield substantially higher precision at roughly equivalent recall for all ncRNA classes tested, taking either an overall average or a median of class averages (Table 2). Our "zRNAfold (Dual)" implementation is conceptually similar to "Dynalign" used by Uzilov [7] (and is related to FoldAlign [14] in that the Z-score is derived from the sum of the stabilities and the variance obtained from shuffle-pair.pl). In our hands Dynalign was too slow (>100 times slower than zMFOLD and zRNAfold) to be useful for genome-wide scanning (our test set alone would require ~1 year of CPU time, or approximately 50–500 times longer than for the other algorithms we tested) and we thus did not test it.

**Figure 3:**
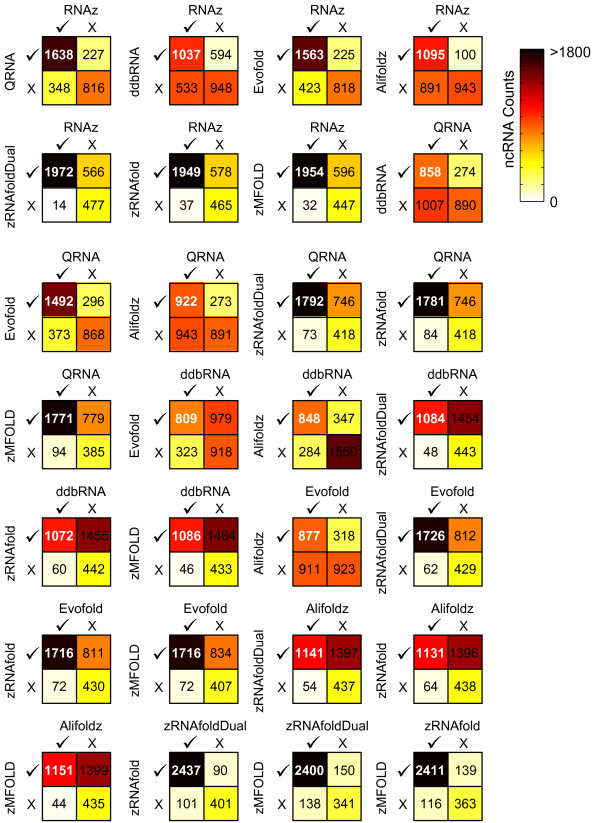
**Pairwise comparisons of positive test ncRNAs identified by different search tools**. McNemar's analysis: counts of correct () and incorrect () classifications of 3046 ncRNAs from pairwise combinations of tools using optimal score thresholds for each tool determined from the score resulting in a maximum sensitivity × specificity (TN/(TN + FP)) for scores combined across all sequence identities.

A less expected but intriguing result was that tools utilizing apparently unrelated algorithms often mutually identified and missed the same ncRNAs (Figure 3). For example, Evofold, one of the covariance tools, displayed a very significant overlap in true positives with all of the thermodynamic-only tools (P ≤ 10^-111^, hypergeometric distribution vs. zRNAfold (dual)).

### Importance of dinucleotide frequency

A somewhat discouraging outcome of our test was that all of the tools displayed low apparent precision in our task. At 50% recall, all of the tools displayed between 1 and 20% precision (median 5.5%). In a genomic scan, in which the background sequence most likely exceeds the 100× ratio we employed (i.e. it is likely that less than 1% of the alignable portions of the genome encode functional RNA structures), this would translate to laboratory confirmations being overwhelmingly negative, in the absence of further filtering. The results of our analysis must be viewed in light of being based only on pairwise alignments, which substantially handicaps tools such as ddbRNA, MSARI, and Evofold that rely solely on covariance and were created solely to scan multiple alignments. Nonetheless, we were surprised that RNAz, which has both covariance and thermodynamic components [10], did not generally outperform zRNAfold (dual), which is our relatively simple two-strand Z-score implementation of the RNAz thermodynamic input.

We reasoned that retention of dinucleotide counts might account for the high false-positive rate we observed. To ask whether this is the case, we examined the results obtained using a negative-control test set generated using an alternative shuffling procedure, shuffle-aln.pl [18], which preserves local conservation, gap structure, and base composition, but not dinucleotide content. Indeed, with shuffle-aln.pl, the apparent false-positive rate (number of negative-control sequences exceeding a threshold) decreased 1.7-fold on average; most drastically for RNAz and QRNA, which dropped 2.9 and 3.1-fold, respectively, at 50% recall (Table 2). This indicates that virtually all tools, including those that do not explicitly have a thermodynamic component, are nonetheless influenced in some way by dinucleotide counts, which it is known should be accounted for in estimation of false-positive rates based on thermodynamic stability [8].

### Considerations in genomic scanning: dinucleotides and other aspects of sequence content

**Figure 4:**
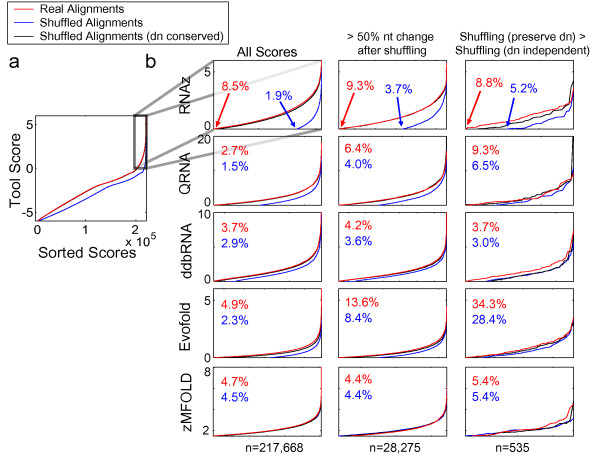
**Dinucleotide frequencies affect assessment of false discovery in genomic tiling**. **a) **Sorted scores generated by tiling human-mouse UCSC chromosome 19 alignments (hg17-mm6) in 120 nt windows beginning every 40 nts (for minimum aligned blocks of length 200 nt) (red line). Each window was also scored after shuffling with shuffle-pair.pl (preserves dinucleotides; black line) and shuffle-aln.pl [18] (which does not preserve dinucleotides; blue line). **b) **Distributions of scores > 0. The left panels show all scores. To minimize the impact of lower "shufflability" (resulting from the additional constraint of preserving dinucleotides) as contributing to higher scores, the plot was also generated limited to sequences where both shuffling methods changed a minimum of 50% nucleotide identities (middle panels), and to sequences where shuffling while conserving dinucleotides exceeded regular shuffling (right panels). Percentages shown in the panels represent the proportion of all tiling windows scoring above the threshold shown.

We next explored whether the dinucleotide issue might influence genomic scans. We ran five of the eight tools on 120-base tiling windows of human-mouse alignments of chromosome 19 (most of which is believed not to be under selection [19]), and compared the distributions of scores with and without preservation of dinucleotide content, again by comparing shuffle-pair.pl with shuffle-aln.pl). (Due to computational constraints, zRNAfold, zRNAfold (dual), and Alifoldz were not run on these sequences). In this analysis, the estimated false-positive rates drastically increased when using shuffles with conserved dinucleotides: in Figure 4, the scores obtained from shuffled sequences with dinucleotide frequency preserved (black line) nearly overlap those obtained from the original sequence (red line), whereas there is a clear separation of both from the scores with mononucleotide shuffling (blue line). We reasoned that insufficient shuffling (due to the dinucleotide preservation constraint) might provide a trivial explanation for this observation; however, when we subset the analysis to those shuffles which differed in at least 50% of their nucleotide positions from their original sequences, the trend clearly remained (Figure 4B, centre column). Even among the 535 windows in which, by chance, the degree of shuffling in the dinucleotide-constrained set exceeded that in the mononucleotide-shuffled set, the trend remained largely intact (these tend to represent the most highly-conserved sequence windows and may represent special cases) (Figure 4B, right column).

**Figure 5:**
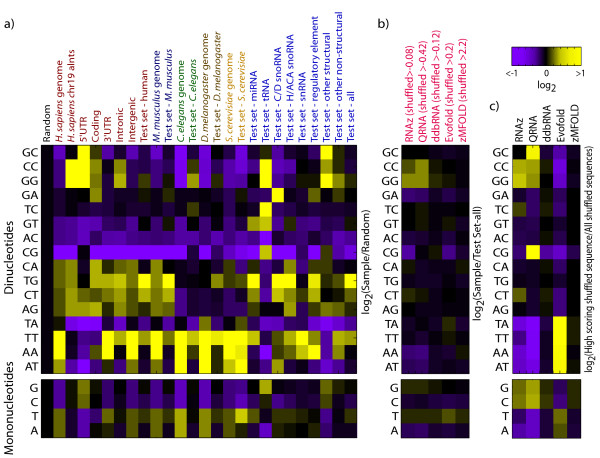
**Relative di- and mononucleotide counts in genomic, test, and positively-scoring shuffled sequences**. **a) **Dinucleotide frequency enrichments over random sequence (where frequency of each dinucleotide is 1/16) for five genomes, human genomic features, and the ncRNA test set subdivided by species and ncRNA classes. Frequency was computed by counting each of the 16 dinucleotides across the indicated sequences and dividing by the total count of all dinucleotides. The bottom panel indicates enrichment of individual nt over random sequence (where frequency is 1/4). **b) **Dinucleotide enrichments in high-scoring shuffled ncRNA alignments (i.e. the test set, frequencies derived form top-strand) for five ncRNA search tools. Frequencies were normalized to the ncRNA test set. Score thresholds were selected to maximize (precision × recall) on our test set (threshold values are indicated in parentheses). **c) **Dinucleotide enrichments among the human strand of 1,000 highest-scoring sequences in shuffled Chromosome 19 genomic alignments (tiled in 120 nt windows every 40 bases).

**Figure 6:**
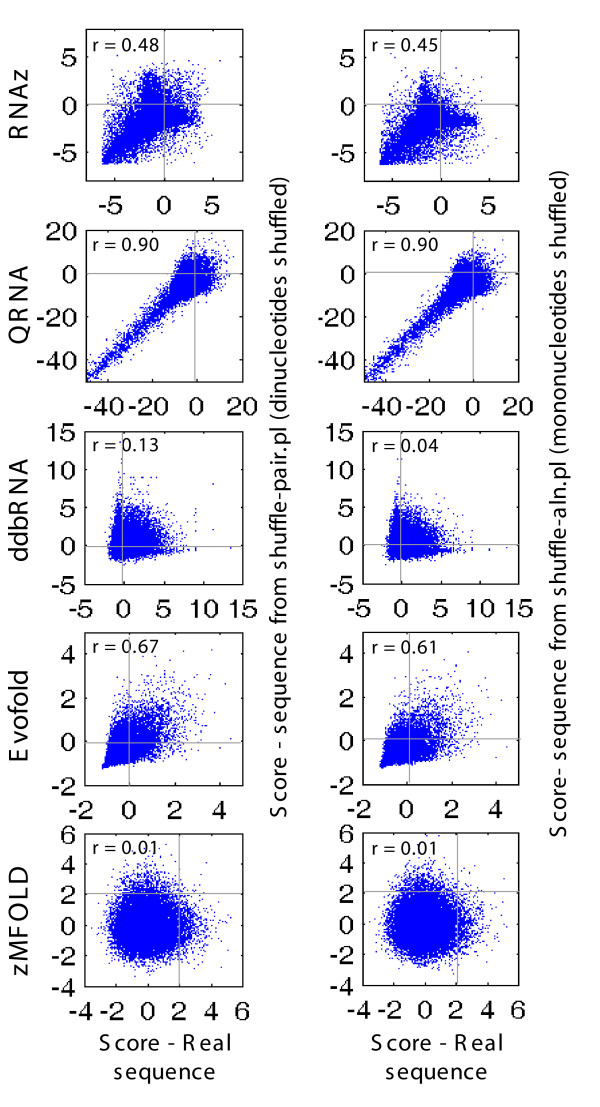
**Relationship between scores of real and shuffled genomic windows**. Each scatter plot shows scores for 28,275 real tiling windows from Chromosome 19 in which more than 50% of all nucleotides were replaced in dinucleotide shuffling by shuffle-pair.pl (the same 28,275 as in the middle column of Figure 4b). In all cases the horizontal axis is real sequence, vertical axis is shuffled. *left*, shuffling using shuffle-pair.pl; *right*, using shuffle-aln.pl.

Why do dinucleotide counts influence scores from these tools? For those relying on thermodynamics (zMFOLD and RNAz), the dependence of Δ*G *on dinucleotides provides a likely explanation. However, QRNA does not utilize thermodynamic stability. Indeed, all the tools we examined displayed negligible biases towards specific nucleotides or dinucleotides when run on randomly-generated alignments (data not shown). Only on ncRNAs and genomic sequence (real or shuffled), which contain non-random distributions of di- and even mononucleotides (Figure 5A), did high-scoring  sequences emerge with enrichment of specific dinucleotides. For example, in shuffled versions of the test set, the highest-scoring sequences from RNAz and QRNA tended to be slightly enriched for CC and GG, and depleted of AA and AT (Figure 5B). This trend was even more marked for shuffled versions of Chromosome 19 alignments (Figure 5C). EvoFold appeared to be even more sensitive to dinucleotide composition of the shuffled genomic alignments, favoring those with AA, TT, AT, and TA (Figure 5C). Manual examination of the specific shuffled genomic sequences that scored highly with EvoFold revealed that many of them contained unusual distributions of homopolymeric or near-homopolymeric runs and simple repeat-like sequence (missed by RepeatMasker) that are extremely rare in randomly-generated sequence, but very common in the human genome. Because the mono-, di-, and/or tri-nucleotide contents have reduced complexity, these patterns are occasionally preserved in shuffled controls. In fact, we observed striking correlations between scores for all of the Chromosome 19 tiling windows before and after shuffling (either mono- or di-nucleotides) (Figure 6), suggesting that simple sequence properties can and do influence scores emerging from these tools.

In an effort to gain further insight, we examined the highest-scoring ~1,500 real genomic sequences from Chromosome 19 for each of three tools utilizing covariance (EvoFold, QRNA, and RNAz). These are posted in the Supplementary data [see Additional file 9]. Manual examination of all three lists revealed the presence of many homopolymeric or near-homopolymeric runs, repetitive short motifs that escape repeatmasking, and nonuniform distribution of nucleotides. The sequences that RNAz and QRNA scored most highly were clearly enriched for G and C (G or C content of ~60% for RNAz; ~66% for QRNA) while those scored most highly by EvoFold appeared enriched for homopolymeric stretches of A and T (and on the whole are only ~42% G or C) (Sequences scored highly by zMFOLD contained ~50% G or C, as did the input alignments). Anecdotally, these non-repeat-masked features do appear to contribute to the predicted RNA structures. While we cannot rule out that a subset of these are *bona fide *undiscovered functional ncRNA features, it is also possible that the non-random distribution of nucleotides and short sequence features in genomes (including short repeats and palindromes presumably arising from aberrant DNA replication and repair events) could lead to a higher degree of apparent secondary structure than would be expected from a completely random evolutionary process.

We currently have no unifying explanation for why these tools appear to favour specific types of sequences, and suggest that this subject might form the basis for further study. These trends do seem to suggest that caution should be exercised in drawing conclusions about the number of ncRNA features in the human genome, on the basis of results of computational genomic scans. From our analyses, we conclude that an accurate assessment of state-of-the-art ncRNA search tools on multiple alignments, which was our initial objective, is not realistic in the absence of a shuffling algorithm that retains at least an approximation of both dinucleotide frequencies and real alignment structure. Moreover, it may be worth considering the fact that genome sequences contain a non-random distribution of mononucleotides and other short sequence features.

### Genomic scanning for novel conserved RNA structural elements reveals enrichment for stable secondary structure in intergenic regions, introns, and UTRs, and selection against structure in coding regions

Our analysis above indicates that with any of the tools we tested, the vast majority of high-scoring windows in a genome scan using pairwise alignments would be false-positives. Nonetheless, we next asked whether use of shuffle-pair.pl, which we believe provides a realistic estimate of the false-positive rate, could aid in making new observations regarding frequency of structural elements across genomic features. We used a tiling strategy similar to that employed previously [10,20,21]. We used a window size of 75 nt (at 30 nt offsets) because this window size resulted in the greatest sensitivity at little cost to specificity relative to other tiling window sizes (data not shown). This window size is within range of previously computed genomic scans: 50 [20] and 200 [21] used in bacterial scans, and 120 [10] used for the human genome. On average, 23% of nucleotides in a 75 base window of human-mouse pairwise alignments change identity during shuffling; however, 13% of sliding windows have >50% shuffling.

**Figure 7:**
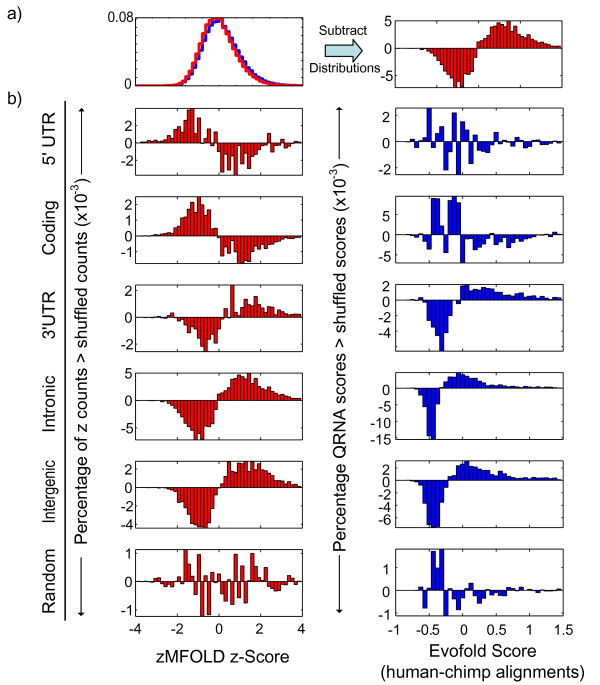
**Enrichment for conserved secondary structure elements across genomic features**. **a) **Differences between real and shuffled score distributions over genomic features. *Left*, Histogram showing distribution of all scores from real and shuffled tiling windows of genomic sequences (see Methods). *Right*, histogram showing the difference between the number of real sequences scoring in a given range and the number of shuffled sequences scoring in the same range. **b) **Histograms showing real-minus-shuffled distributions (as in the right part of panel a of this figure) for different categories of genomic features: *left*, zMFOLD (human vs. mouse); *right*, Evofold (human vs. chimp).

We scanned pairwise genomic alignments [22] of human chromosome 19 and genomic regions corresponding to alignable portions of 24,576 Refseq [23] genes with either zMFOLD or Evofold, as practical representatives of the thermodynamic-stability-only regime and the covariance regime. We scanned real and shuffled counterparts (using shuffle-pair.pl) of each window. We found that the distributions of the real and  shuffled scores largely overlap, with few or no real scores completely exceeding the shuffled distribution (Figure 7a, left). However, the real and shuffled score distributions were significantly different (Wilcoxon rank-sum test, p < e^-100^; data not shown). A histogram of the difference in number of real and shuffled sequences at different scores is shown at the right in Figure 7a. These results obviously bring into question the significance of any individual element, but also suggest a selection for some level of conserved secondary structure across the genome.

To ask whether results obtained from genomic scanning appear to be biologically relevant on the whole, and to gain evidence that we have not mis-calibrated the negative controls (thus under- or overestimating the false-positive rate), we examined the overall distribution of score differences between real and shuffled sequences obtained from different types of genomic features (UTRs, introns, intergenic sequences, and coding regions), which might be expected to display differing propensities towards secondary structure. Using either zMFOLD or Evofold, we found that striking differences exist among results obtained from these features (Figure 7b; note that EvoFold human-chimp comparisons are substituted for human-mouse, as these resulted in a larger number of positives, to our surprise). In particular, introns, intergenic sequence and 3' UTRs tend to have a higher distribution of scores, indicating enrichment for structural features, relative to shuffled controls. In contrast, we observed marginal enrichment within 5' UTRs. Strikingly, we found that coding regions have a lower score distribution than shuffled controls, indicating an overall depletion of RNA structures in open reading frames, which is consistent with the possibility that these elements would inhibit translation [24]. These results indicate that our analysis is not, on average, biased towards falsely reporting structures, since some genomic features appear to be enriched for structures while others are depleted or neutral. These results also provide evidence for the presence of biologically functional structural features, although specificity limitations of the individual tools preclude direct prediction of individual structural elements.

**Figure 8:**
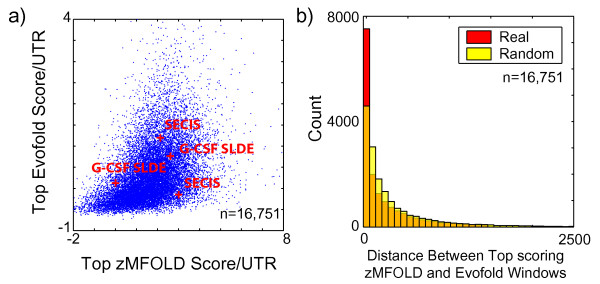
**zMFOLD and Evofold scores from UTRs are related**. **a) **Top Evofold and zMFOLD scores per 3'UTR (normalized to the length of the UTR) are correlated (Pearson 0.43). Selected known 3' UTR structures are indicated in red. **b) **Histograms showing the distance between the top-scoring EvoFold and zMFOLD sequence windows for 3' UTRs analyzed. For the first bar, red + orange = real windows; orange = randomly-selected windows; for the remainder, yellow + orange = randomly-selected windows; orange = real windows.

To further investigate whether the tools are in fact identifying a biologically meaningful selection for structural elements we asked whether zMFOLD and Evofold, which represent very different approaches, tend to give high scores to the same UTR segments. We found that in 3'UTRs, where an  obvious enrichment for structural elements exists, the top zMFOLD and Evofold scores are correlated (i.e. if a 3'UTR has a high scoring Evofold score, it is also likely to contain a high-scoring zMFOLD score; Figure 8A; scores were normalized to UTR length). Furthermore, 48% of Evofold and zMFOLD highest scores within each UTR correspond to the same region of the UTR (within 100 nt of each other) which is approximately two-fold higher than expected by chance (Figure 8B).

## Discussion

To our knowledge, the panel of ncRNA search tools examined here have not been examined systematically by an independent laboratory. With the goal of evaluating how useful these tools are for *de novo *discovery of ncRNA elements in a genomic context, in which the anticipated sparseness of real features makes it particularly important to control false discovery, we compiled an extensive sequence and alignment data set comprised of 3046 fragments of genomic alignments [22] corresponding to ncRNAs from a variety of eukaryotes. We also developed a shuffling algorithm for pairwise alignments (shuffle-pair.pl) that maintains dinucleotide frequencies, in addition to other features typically maintained in alignment shuffling (gap structure, base composition, and local conservation) [10,16].

Due to shuffling constraints, which we show are important, we limited our test to pairwise alignments. In our test, we found that failure to account for background sequence properties leads to over-estimation of precision (i.e. under-estimation of false-positive rate) for most tools. We were surprised to find that tools with a strong or exclusive covariance component were sensitive, in at least some situations, to  dinucleotide counts, which should primarily impact thermodynamic stability. Estimation of background rates from shuffling procedures that do not preserve dinucleotides in our study resulted in an underestimate of the false-positive rate of up to 3-fold in our analysis. We also confirmed that, for pairwise alignments, *z*-scores derived from assessing thermodynamic stability (relative to stabilities calculated from the same sequences run through shuffle-pair.pl) contained comparable discriminatory potential to the best alignment-based tools applied to pairwise alignments, on the basis of precision at comparable recall.

Although our shuffling constraints precluded analysis of more than pairwise alignments, it is possible that the false-positive rates of these tools on multiple alignments are higher than has been previously estimated, as their false-positive rates are usually estimated by shuffling mononucleotides [16] or by simulating evolution without conserving dinucleotides [13]. For RNAz, a possible explanation is that the score incorporates thermodynamic stability which is calculated by sampling from precomputed stabilities from sequences with similar length and mononucleotide composition (not dinucleotide composition or any other aspects of the sequence). The SVM sampling could be extended to include a dinucleotide sampling which would likely alleviate any dinucleotide bias. We are less certain of the explanation for the apparent dinucleotide biases evident for QRNA (for both our test set and our Chromosome 19 scan) and Evofold (for the Chromosome 19 scan). It seems unlikely to be a result of dinucleotide bias *per se *although QRNA may have learned a GC bias from tRNAs in its test set. We propose that these algorithms may suffer from not having been exposed to a large amount of nonselected mammalian genomic sequence as negative controls, as genomic sequence is known to contain substantial local variation in nucleotide content as well as a non-random distribution of other simple sequence features [25].

Our results underscore the importance of background modelling, which we believe to be one of the major unresolved issues in *de novo *ncRNA searching in practice. Although our demonstration is based on pairwise alignments, multiple alignments should not be immune from these same trends. Unfortunately, the difficulty of preserving dinucleotides while shuffling increases substantially as the number of sequences in the alignment increases, even in the absence of other shuffling constraints.

We see no clean solution to this dilemma, although general strategies can be envisioned. First, the shuffling problem itself deserves more detailed investigation. Our algorithm is only a simple heuristic and it might be improved by finding more efficient ways to explore the space of permutations. Another possibility might be to relax the constraint that every dinucleotide count in every alignment must be preserved. This could be done by repeatedly shuffling the multiple alignment while keeping track of dinucleotide content until a shuffled variant sufficiently close to the native alignment is attained. Pollard et al. [26] generated alignments *in silico *by simulating evolutionary sequence divergence; this method might be adapted to produce similar overall dinucleotide counts and alignment structures to test sequences. An additional possibility is to use longer shuffling windows to shuffle multiple alignments. However, most known structural ncRNAs are relatively short (the median length of ncRNAs in our test set is 82 nt), and precision and recall decrease substantially with tiling window sizes exceeding 150 nt (data not shown). Unfortunately, Altschul-and-Erickson-type shuffling [27], which retains dinucleotide counts in individual sequences but not the alignment structure, is inappropriate even for thermodynamic tools that do not depend on conserved structure for their overall score (such as zRNAfold-Dual). This is because independently shuffling the sequences within the alignment eliminates the dependency of the score contributions from the individual sequences to the overall score, and lowers the range of background scores (up to 2-fold for perfectly conserved alignments). Independent shuffling is clearly inappropriate for tools that require conservation of structure.

An additional issue is that covariance-based tools are reliant on correct alignment as it is assumed that the covarying bases are structurally paired. Unlike protein coding regions, where independent structural evidence often exists to validate sequence alignments, aligning non-coding regions presents additional challenges. Pollard et al. [26] reported that local alignment tools such as blastz (which was used to generate the alignments used in this study) had higher specificity but lower sensitivity than global alignment tools (such as ClustalW) again using simulated evolutionary sequence divergence as a control for background. This implies that although full genome alignments represent orthologous sequences, the exact nucleotide positions may not always be accurate, and thus may not provide an accurate covariance signature.

### Selection pressure to maintain or avoid conserved secondary structure depends on genomic location

Despite these caveats, comparisons of the aggregate scores for real and shuffled alignments among different types of genomic features, including coding and non-coding parts of the genome, revealed striking differences with consequent biological implications. In general, coding sequences appear to possess lower propensity for conserved secondary structure than would be expected by chance. This is not an artefact of the high conservation of coding sequences; highly conserved sequences in fact tend to shuffle more completely (because a string of exact matches eliminates the local conservation shuffling constraint) which would aid in discriminating bona fide structures. On the basis of this result, it seems unlikely that we are dramatically overestimating the degree of selected secondary structure in the genome because at least one type of genomic feature scores as containing less than would be expected by chance. Since the random alignments exactly match the background expectation (Figure 7b), we conclude that there is likely to be an evolutionary selection against formation of secondary structures in open reading frames. To our knowledge, this trend has not yet been demonstrated on a genome-wide scale, although previous experimental data has shown that the introduction of stable secondary structure can indeed impact on translation efficiency [28]. The most obvious rationale for such a selection is that inhibition of translation is, on the whole, detrimental to fitness of the organism. In contrast, we find evidence that 3'UTRs, introns, and intergenic regions on the whole contain slightly higher structural scores than corresponding shuffles. Although the biological significance of all but a small fraction of the highest-scoring individual sequences is questionable, our results does nevertheless support the existence of many additional RNA structural elements in UTRs and introns, and potentially additional classes of ncRNAs in intergenic regions.

## Conclusion

Accurate prediction of RNA structural elements in aligned genome sequence remains a difficult problem. Further work is required to properly assess background detection rates. Nonetheless, the general trends we observed in genomic scans appear to roughly reflect expectations, supporting the presence of secondary structural elements in UTRs, and providing compelling evidence for evolutionary selection against secondary structures in coding regions.

## Methods

### Test set

We downloaded tRNA and miRNA sequences from the tRNA database [29] and Rfam [30] respectively, and all other ncRNAs from NONCODE [31] (March 1, 2005). We downloaded human-mouse pairwise alignments (hg17-mm6) and human Refseq and ENSEMBL gene tracks from UCSC [22] (Feb. 27, 2005). We mapped ncRNAs to the genome using BLAT [32]. We did not consider sequences that did not map in their entirety to an aligned block. We developed Perl scripts to process the data: extract_axt.pl [see Additional file 5] and extract_maf.pl [see Additional file 6] for extraction of relevant pairwise and multiple alignments using genomic coordinates as input (BED format) while taking orientation into account, and calc_cons.pl to extract average PhastCons scores. We generated the ncRNA alignment test set, consisting of 3,046 ncRNA alignments, using pairwise alignments of the reference genome containing the transcript of interest versus several available aligned genomes on the UCSC genome browser. Only unique alignments were retained (i.e. instances where both strands were identical were removed). The test set is available as Additional files 7 and 8, and at [17].

### Shuffling

We carried out shuffling of pairwise alignments using a Perl script, shuffle-pair.pl [see Additional file 3]. shuffle-pair is an extension of the shuffling principle used by Workman and Krogh [8] where a randomly selected trinucleotide is swapped with another randomly selected trinucleotide with identical bases at positions 1 and 3, and this is repeated through *N *iterations. Controlled shuffling of pairwise alignments requires three additional constraints: 1) simultaneous conservation of dinucleotides in both sequences, 2) conservation of sequence identity (i.e. matches and mismatches), and 3) conservation of gaps. shuffle-pair attempts to swap each position in the alignment with randomly selected positions that satisfy these criteria. Beginning with the aligned trinucleotide at position 1 of the alignment, it identifies all other aligned trinucleotides where positions 1 and 3 are identical and where position 2 has identical sequence identity (i.e. match or mismatch). It then randomly selects one of these trinucleotide positions and swaps the columns at position 2. shuffle-pair repeats this for all trinucleotides within the alignment and keeps a record of positions that were swapped. It repeats the overall process until no more swappable positions exist. To prevent disruption of gaps, it treats sequences aligned to gaps as sub-sequences of the overall alignment and shuffles them separately using the process described above.

Pseudocode for shuffle-pair.pl is as follows:

shuffle-pair pseudocode

load in pairwise sequence alignment (seq1, seq2)

binary_conservation_string = find_matches(seq1, seq2)

initialize(swapped_positions)

# shuffle regions that do not contain gaps

do      {

      for i = 1:length(seq1)-3

         if (i+1) is in swapped_positions, skip, i = i+1

         initialize(swappable_positions)

         # idenify all swappable positions

         for j = 1:(length(seq1)-3)

            if   (

               seq1(i) equals seq1(j) &

               seq2(i) equals seq2(j) &

               seq1(i+2) equals seq1(j+2) &

               seq2(i+2) equals seq2(j+2) &

               binary_cons_string(i) equals binary_cons_string(j) &

               binary_cons_string(i+1) equals binary_cons_string(j+1) &

               binary_cons_string(i+2) equals binary_cons_string(j+2) &

               none of these match gap characters &

               (j+1) is not already in swapped_positions

               )

            do   {store(j+1) in swappable_positions}

         end for

         # randomly swap

         swap = swappable_positions(random)

         swap seq1(i+1) and seq2(i+1) with swap

         store swap in swapped_positions

      end for

   }

until { no more swaps occur in full scan of alignment }

# shuffle gapped regions (i.e. gap length > 3)

identify gaps

repeat steps above swapping only in gapped regions

### Running ncRNA-finding tools

We ran MSARI, RNAz, ddbRNA, QRNA (v.2), and Evofold on the real and shuffled test sets using default parameters. We used MFOLD and other thermodynamics tools to compute *z*-scores, where *z *= -(Δ*G*_real sequence _- mean(Δ*G*_100 shuffled sequences_)/StandardDev_shuffled score distribution_. Briefly, *z *is the number of standard deviations that the true stability (of the single reference strand) is below the mean stability of the shuffled sequences (higher z-scores indicate higher stability over shuffles; in our implementation 100 shuffles generated using shuffle-pair.pl). A script that calculates thermodynamic stability  z-score using MFOLD is given in Additional file 4.

### Generation of random alignments

We generated random alignments on a per-column basis using real alignments as input. Each nucleotide was replaced using a random alphabet map that was regenerated for each column. The random alphabet map consisted of the assignment of A, C, G, and T to a random order of A, C, G, and T (e.g. ACGT to TGCA, so that all As would be replaced with Ts, Cs with Gs, etc.). Gaps were maintained, to produce alignments with the same conservation pattern and gap structure as the input alignments, but with a different base composition.

### Genomic sequences

We extracted intronic, coding, and UTR alignments from human-mouse pairwise alignments [22] using extract-axt.pl [see Additional file 5] using Refseq coordinates (UCSC Table Browser). We extracted intergenic alignments from chromosome 19 pairwise alignments using coordinates that did not overlap with Refseq genes, human mRNA, and Known Genes [22] in any orientation.

### Data Availability

The shuffling algorithm implemented in Perl (shuffle-pair.pl) and all supplementary files are available as additional files and at the author's website [17].

## Authors' contributions

TB carried out the data analysis. BB and TH coordinated the study. All authors contributed to preparation of the manuscript.

## Supplementary Material

Additional File 7 **ncRNA set**. Set of human, mouse, fly, worm, and yeast ncRNAs used to compile the test set. Click here for file

Additional File 8 **ncRNA alignment test set**. 3046 eukaryotic ncRNA pairwise alignments. Click here for file

Additional File 3 Shuffles pairwise alignments while preserving dinucleotides (in both sequences), base composition, gaps, and local conservation. Click here for file

Additional File 1 **Precision-recall plots for individual ncRNA classes (Supplementary Figures 1 through 8)**. Dependency of precision and recall on score threshold and level of conservation (as per Figure 2). Click here for file

Additional File 2 **Supplementary Table 1**. Detailed results on test set for EvoFold with and without members of the EvoFold training set. Click here for file

Additional File 9 **high-scoring sequence windows from chromosome 19**. Human strands shown. Some are shorter than 120 bases due to the presence of gaps in the human genome sequence. Click here for file

Additional File 5 Extracts portions of UCSC pairwise genomic alignments. Takes as input genomic coordinates (BED file format) and alignment files and outputs alignments for coordinates where alignments exist. Click here for file

Additional File 6 Extracts portions of UCSC multiple genomic alignments (human 8-way). Takes as input human genomic coordinates (BED file format) and alignment files and outputs alignments where possible (i.e. alignments exist). Click here for file

Additional File 4 Script that calculates thermodynamic stability z-score using MFOLD (see Table 1). Click here for file

## References

[B1] Waterston RH, Lindblad-Toh K, Birney E, Rogers J, Abril JF, Agarwal P, Agarwala R, Ainscough R, Alexandersson M, An P, Antonarakis SE, Attwood J, Baertsch R, Bailey J, Barlow K, Beck S, Berry E, Birren B, Bloom T, Bork P, Botcherby M, Bray N, Brent MR, Brown DG, Brown SD, Bult C, Burton J, Butler J, Campbell RD, Carninci P, Cawley S, Chiaromonte F, Chinwalla AT, Church DM, Clamp M, Clee C, Collins FS, Cook LL, Copley RR, Coulson A, Couronne O, Cuff J, Curwen V, Cutts T, Daly M, David R, Davies J, Delehaunty KD, Deri J, Dermitzakis ET, Dewey C, Dickens NJ, Diekhans M, Dodge S, Dubchak I, Dunn DM, Eddy SR, Elnitski L, Emes RD, Eswara P, Eyras E, Felsenfeld A, Fewell GA, Flicek P, Foley K, Frankel WN, Fulton LA, Fulton RS, Furey TS, Gage D, Gibbs RA, Glusman G, Gnerre S, Goldman N, Goodstadt L, Grafham D, Graves TA, Green ED, Gregory S, Guigo R, Guyer M, Hardison RC, Haussler D, Hayashizaki Y, Hillier LW, Hinrichs A, Hlavina W, Holzer T, Hsu F, Hua A, Hubbard T, Hunt A, Jackson I, Jaffe DB, Johnson LS, Jones M, Jones TA, Joy A, Kamal M, Karlsson EK, Karolchik D, Kasprzyk A, Kawai J, Keibler E, Kells C, Kent WJ, Kirby A, Kolbe DL, Korf I, Kucherlapati RS, Kulbokas EJ, Kulp D, Landers T, Leger JP, Leonard S, Letunic I, Levine R, Li J, Li M, Lloyd C, Lucas S, Ma B, Maglott DR, Mardis ER, Matthews L, Mauceli E, Mayer JH, McCarthy M, McCombie WR, McLaren S, McLay K, McPherson JD, Meldrim J, Meredith B, Mesirov JP, Miller W, Miner TL, Mongin E, Montgomery KT, Morgan M, Mott R, Mullikin JC, Muzny DM, Nash WE, Nelson JO, Nhan MN, Nicol R, Ning Z, Nusbaum C, O'Connor MJ, Okazaki Y, Oliver K, Overton-Larty E, Pachter L, Parra G, Pepin KH, Peterson J, Pevzner P, Plumb R, Pohl CS, Poliakov A, Ponce TC, Ponting CP, Potter S, Quail M, Reymond A, Roe BA, Roskin KM, Rubin EM, Rust AG, Santos R, Sapojnikov V, Schultz B, Schultz J, Schwartz MS, Schwartz S, Scott C, Seaman S, Searle S, Sharpe T, Sheridan A, Shownkeen R, Sims S, Singer JB, Slater G, Smit A, Smith DR, Spencer B, Stabenau A, Stange-Thomann N, Sugnet C, Suyama M, Tesler G, Thompson J, Torrents D, Trevaskis E, Tromp J, Ucla C, Ureta-Vidal A, Vinson JP, Von Niederhausern AC, Wade CM, Wall M, Weber RJ, Weiss RB, Wendl MC, West AP, Wetterstrand K, Wheeler R, Whelan S, Wierzbowski J, Willey D, Williams S, Wilson RK, Winter E, Worley KC, Wyman D, Yang S, Yang SP, Zdobnov EM, Zody MC, Lander ES (2002). Initial sequencing and comparative analysis of the mouse genome. Nature.

[B2] Eddy SR (2002). Computational genomics of noncoding RNA genes. Cell.

[B3] Argaman L, Hershberg R, Vogel J, Bejerano G, Wagner EG, Margalit H, Altuvia S (2001). Novel small RNA-encoding genes in the intergenic regions of Escherichia coli. Curr Biol.

[B4] Wassarman KM, Repoila F, Rosenow C, Storz G, Gottesman S (2001). Identification of novel small RNAs using comparative genomics and microarrays. Genes Dev.

[B5] Carter RJ, Dubchak I, Holbrook SR (2001). A computational approach to identify genes for functional RNAs in genomic sequences. Nucleic Acids Res.

[B6] Clote P, Ferre F, Kranakis E, Krizanc D (2005). Structural RNA has lower folding energy than random RNA of the same dinucleotide frequency. Rna.

[B7] Uzilov AV, Keegan JM, Mathews DH (2006). Detection of non-coding RNAs on the basis of predicted secondary structure formation free energy change. BMC Bioinformatics.

[B8] Workman C, Krogh A (1999). No evidence that mRNAs have lower folding free energies than random sequences with the same dinucleotide distribution. Nucleic Acids Res.

[B9] Rivas E, Eddy SR (2001). Noncoding RNA gene detection using comparative sequence analysis. BMC Bioinformatics.

[B10] Washietl S, Hofacker IL, Stadler PF (2005). Fast and reliable prediction of noncoding RNAs. Proc Natl Acad Sci U S A.

[B11] di Bernardo D, Down T, Hubbard T (2003). ddbRNA: detection of conserved secondary structures in multiple alignments. Bioinformatics.

[B12] Coventry A, Kleitman DJ, Berger B (2004). MSARI: multiple sequence alignments for statistical detection of RNA secondary structure. Proc Natl Acad Sci U S A.

[B13] Pedersen JS, Bejerano G, Siepel A, Rosenbloom K, Lindblad-Toh K, Lander ES, Kent J, Miller W, Haussler D (2006). Identification and Classification of Conserved RNA Secondary Structures in the Human Genome. PLoS Comput Biol.

[B14] Havgaard JH, Lyngso RB, Stormo GD, Gorodkin J (2005). Pairwise local structural alignment of RNA sequences with sequence similarity less than 40%. Bioinformatics.

[B15] Torarinsson E, Sawera M, Havgaard JH, Fredholm M, Gorodkin J (2006). Thousands of corresponding human and mouse genomic regions unalignable in primary sequence contain common RNA structure. Genome Res.

[B16] Washietl S, Hofacker IL, Lukasser M, Huttenhofer A, Stadler PF (2005). Mapping of conserved RNA secondary structures predicts thousands of functional noncoding RNAs in the human genome. Nat Biotechnol.

[B17] Tools_Data [http://hugheslab.med.utoronto.ca/Babak/tools/].

[B18] Washietl S, Hofacker IL (2004). Consensus folding of aligned sequences as a new measure for the detection of functional RNAs by comparative genomics. J Mol Biol.

[B19] Siepel A, Bejerano G, Pedersen JS, Hinrichs AS, Hou M, Rosenbloom K, Clawson H, Spieth J, Hillier LW, Richards S, Weinstock GM, Wilson RK, Gibbs RA, Kent WJ, Miller W, Haussler D (2005). Evolutionarily conserved elements in vertebrate, insect, worm, and yeast genomes. Genome Res.

[B20] Katz L, Burge CB (2003). Widespread selection for local RNA secondary structure in coding regions of bacterial genes. Genome Res.

[B21] Rivas E, Klein RJ, Jones TA, Eddy SR (2001). Computational identification of noncoding RNAs in E. coli by comparative genomics. Curr Biol.

[B22] Hinrichs AS, Karolchik D, Baertsch R, Barber GP, Bejerano G, Clawson H, Diekhans M, Furey TS, Harte RA, Hsu F, Hillman-Jackson J, Kuhn RM, Pedersen JS, Pohl A, Raney BJ, Rosenbloom KR, Siepel A, Smith KE, Sugnet CW, Sultan-Qurraie A, Thomas DJ, Trumbower H, Weber RJ, Weirauch M, Zweig AS, Haussler D, Kent WJ (2006). The UCSC Genome Browser Database: update 2006. Nucleic Acids Res.

[B23] Pruitt KD, Tatusova T, Maglott DR (2005). NCBI Reference Sequence (RefSeq): a curated non-redundant sequence database of genomes, transcripts and proteins. Nucleic Acids Res.

[B24] Pelletier J, Sonenberg N (1987). The involvement of mRNA secondary structure in protein synthesis. Biochem Cell Biol.

[B25] Lander ES, Linton LM, Birren B, Nusbaum C, Zody MC, Baldwin J, Devon K, Dewar K, Doyle M, FitzHugh W, Funke R, Gage D, Harris K, Heaford A, Howland J, Kann L, Lehoczky J, LeVine R, McEwan P, McKernan K, Meldrim J, Mesirov JP, Miranda C, Morris W, Naylor J, Raymond C, Rosetti M, Santos R, Sheridan A, Sougnez C, Stange-Thomann N, Stojanovic N, Subramanian A, Wyman D, Rogers J, Sulston J, Ainscough R, Beck S, Bentley D, Burton J, Clee C, Carter N, Coulson A, Deadman R, Deloukas P, Dunham A, Dunham I, Durbin R, French L, Grafham D, Gregory S, Hubbard T, Humphray S, Hunt A, Jones M, Lloyd C, McMurray A, Matthews L, Mercer S, Milne S, Mullikin JC, Mungall A, Plumb R, Ross M, Shownkeen R, Sims S, Waterston RH, Wilson RK, Hillier LW, McPherson JD, Marra MA, Mardis ER, Fulton LA, Chinwalla AT, Pepin KH, Gish WR, Chissoe SL, Wendl MC, Delehaunty KD, Miner TL, Delehaunty A, Kramer JB, Cook LL, Fulton RS, Johnson DL, Minx PJ, Clifton SW, Hawkins T, Branscomb E, Predki P, Richardson P, Wenning S, Slezak T, Doggett N, Cheng JF, Olsen A, Lucas S, Elkin C, Uberbacher E, Frazier M, Gibbs RA, Muzny DM, Scherer SE, Bouck JB, Sodergren EJ, Worley KC, Rives CM, Gorrell JH, Metzker ML, Naylor SL, Kucherlapati RS, Nelson DL, Weinstock GM, Sakaki Y, Fujiyama A, Hattori M, Yada T, Toyoda A, Itoh T, Kawagoe C, Watanabe H, Totoki Y, Taylor T, Weissenbach J, Heilig R, Saurin W, Artiguenave F, Brottier P, Bruls T, Pelletier E, Robert C, Wincker P, Smith DR, Doucette-Stamm L, Rubenfield M, Weinstock K, Lee HM, Dubois J, Rosenthal A, Platzer M, Nyakatura G, Taudien S, Rump A, Yang H, Yu J, Wang J, Huang G, Gu J, Hood L, Rowen L, Madan A, Qin S, Davis RW, Federspiel NA, Abola AP, Proctor MJ, Myers RM, Schmutz J, Dickson M, Grimwood J, Cox DR, Olson MV, Kaul R, Raymond C, Shimizu N, Kawasaki K, Minoshima S, Evans GA, Athanasiou M, Schultz R, Roe BA, Chen F, Pan H, Ramser J, Lehrach H, Reinhardt R, McCombie WR, de la Bastide M, Dedhia N, Blocker H, Hornischer K, Nordsiek G, Agarwala R, Aravind L, Bailey JA, Bateman A, Batzoglou S, Birney E, Bork P, Brown DG, Burge CB, Cerutti L, Chen HC, Church D, Clamp M, Copley RR, Doerks T, Eddy SR, Eichler EE, Furey TS, Galagan J, Gilbert JG, Harmon C, Hayashizaki Y, Haussler D, Hermjakob H, Hokamp K, Jang W, Johnson LS, Jones TA, Kasif S, Kaspryzk A, Kennedy S, Kent WJ, Kitts P, Koonin EV, Korf I, Kulp D, Lancet D, Lowe TM, McLysaght A, Mikkelsen T, Moran JV, Mulder N, Pollara VJ, Ponting CP, Schuler G, Schultz J, Slater G, Smit AF, Stupka E, Szustakowski J, Thierry-Mieg D, Thierry-Mieg J, Wagner L, Wallis J, Wheeler R, Williams A, Wolf YI, Wolfe KH, Yang SP, Yeh RF, Collins F, Guyer MS, Peterson J, Felsenfeld A, Wetterstrand KA, Patrinos A, Morgan MJ, de Jong P, Catanese JJ, Osoegawa K, Shizuya H, Choi S, Chen YJ (2001). Initial sequencing and analysis of the human genome. Nature.

[B26] Pollard DA, Bergman CM, Stoye J, Celniker SE, Eisen MB (2004). Benchmarking tools for the alignment of functional noncoding DNA. BMC Bioinformatics.

[B27] Altschul SF, Erickson BW (1985). Significance of nucleotide sequence alignments: a method for random sequence permutation that preserves dinucleotide and codon usage. Mol Biol Evol.

[B28] Pelletier J, Sonenberg N (1985). Insertion mutagenesis to increase secondary structure within the 5' noncoding region of a eukaryotic mRNA reduces translational efficiency. Cell.

[B29] Sprinzl M, Horn C, Brown M, Ioudovitch A, Steinberg S (1998). Compilation of tRNA sequences and sequences of tRNA genes. Nucleic Acids Res.

[B30] Griffiths-Jones S, Moxon S, Marshall M, Khanna A, Eddy SR, Bateman A (2005). Rfam: annotating non-coding RNAs in complete genomes. Nucleic Acids Res.

[B31] Liu C, Bai B, Skogerbo G, Cai L, Deng W, Zhang Y, Bu D, Zhao Y, Chen R (2005). NONCODE: an integrated knowledge database of non-coding RNAs. Nucleic Acids Res.

[B32] Kent WJ (2002). BLAT--the BLAST-like alignment tool. Genome Res.

[B33] Hofacker IL (2003). Vienna RNA secondary structure server. Nucleic Acids Res.

[B34] Markham NR, Zuker M (2005). DINAMelt web server for nucleic acid melting prediction. Nucleic Acids Res.

[B35] Hofacker IL, Fontana W, Stadler PF, Bonhoeffer LS, Tacker M, Schuster P (1994). Fast Folding and Comparison of RNA Secondary Structures. MonatshChem.

