# Supplementary material for: Considerations in the identification of functional RNA structural elements in genomic alignments
Source: BMC Bioinformatics. 2007 Jan 30;8:33. doi: 10.1186/1471-2105-8-33 (PMC1803800; doi:10.1186/1471-2105-8-33)
Supplement: Additional File 1 — Precision-recall plots for individual ncRNA classes (Supplementary Figures 1 through 8). Dependency of precision and recall on score threshold and level of conservation (as per Figure 2). [file 1471-2105-8-33-S1.pdf]

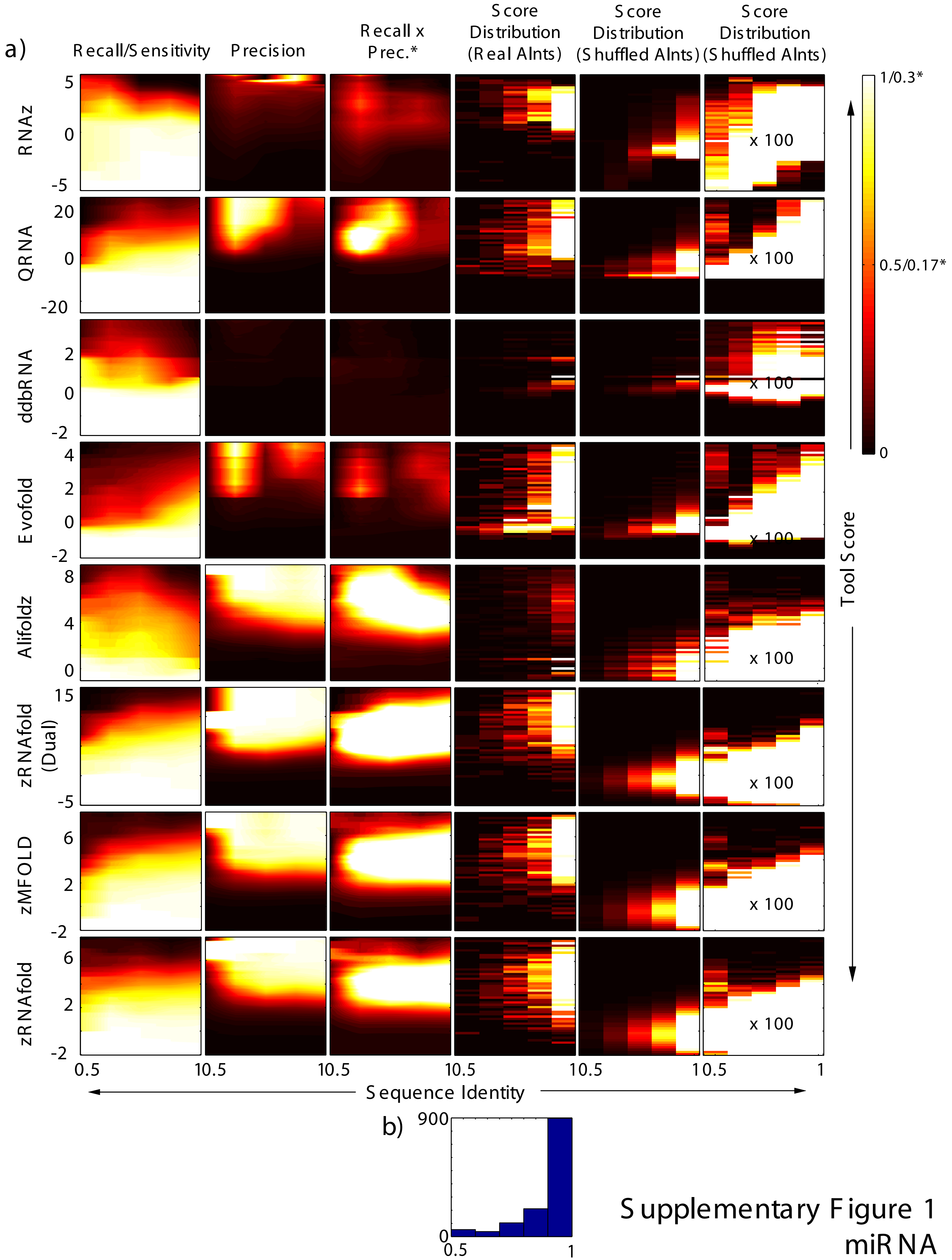

Supplementary Figure 1
miRNA

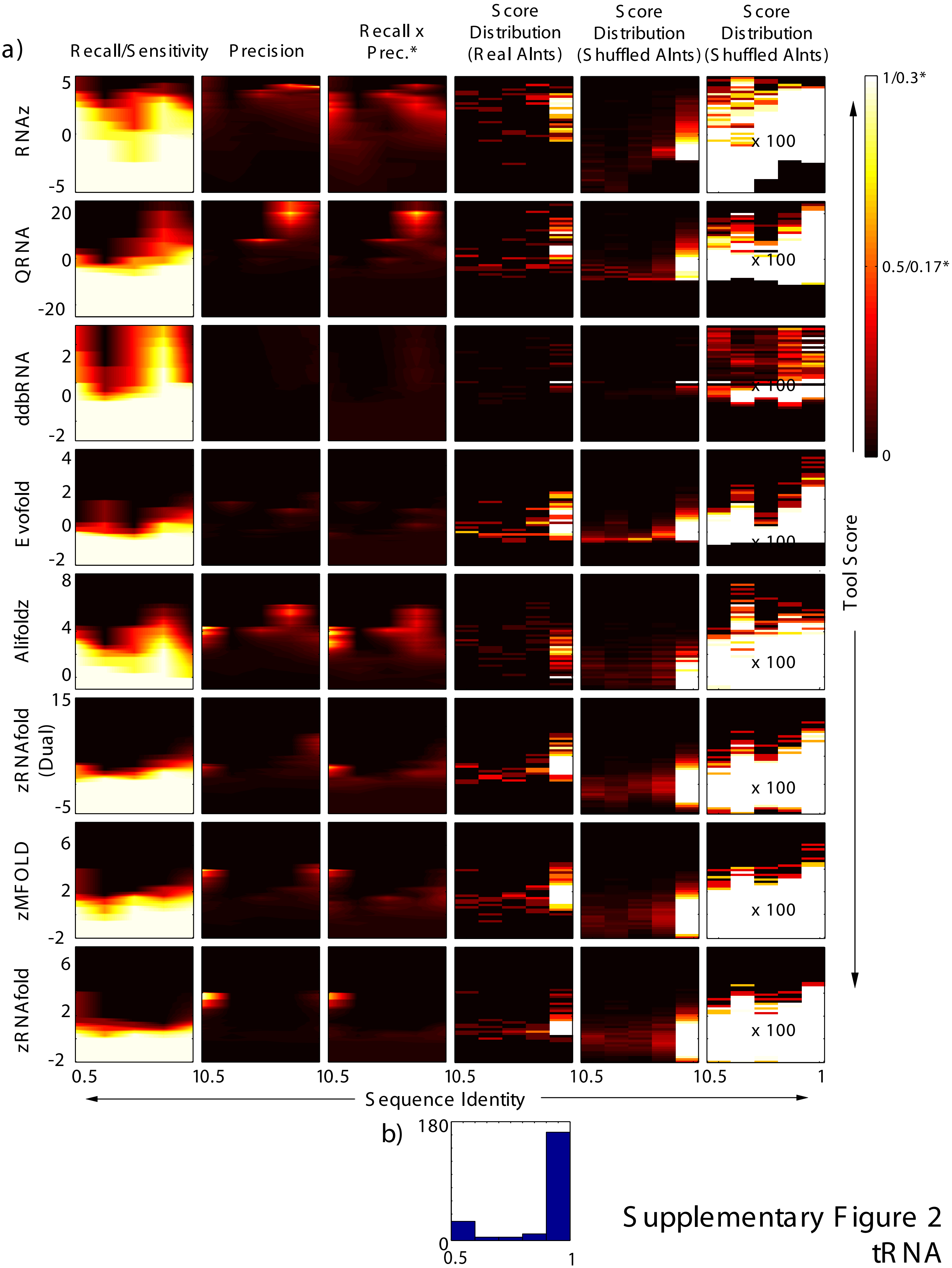

Supplementary Figure 2
tRNA

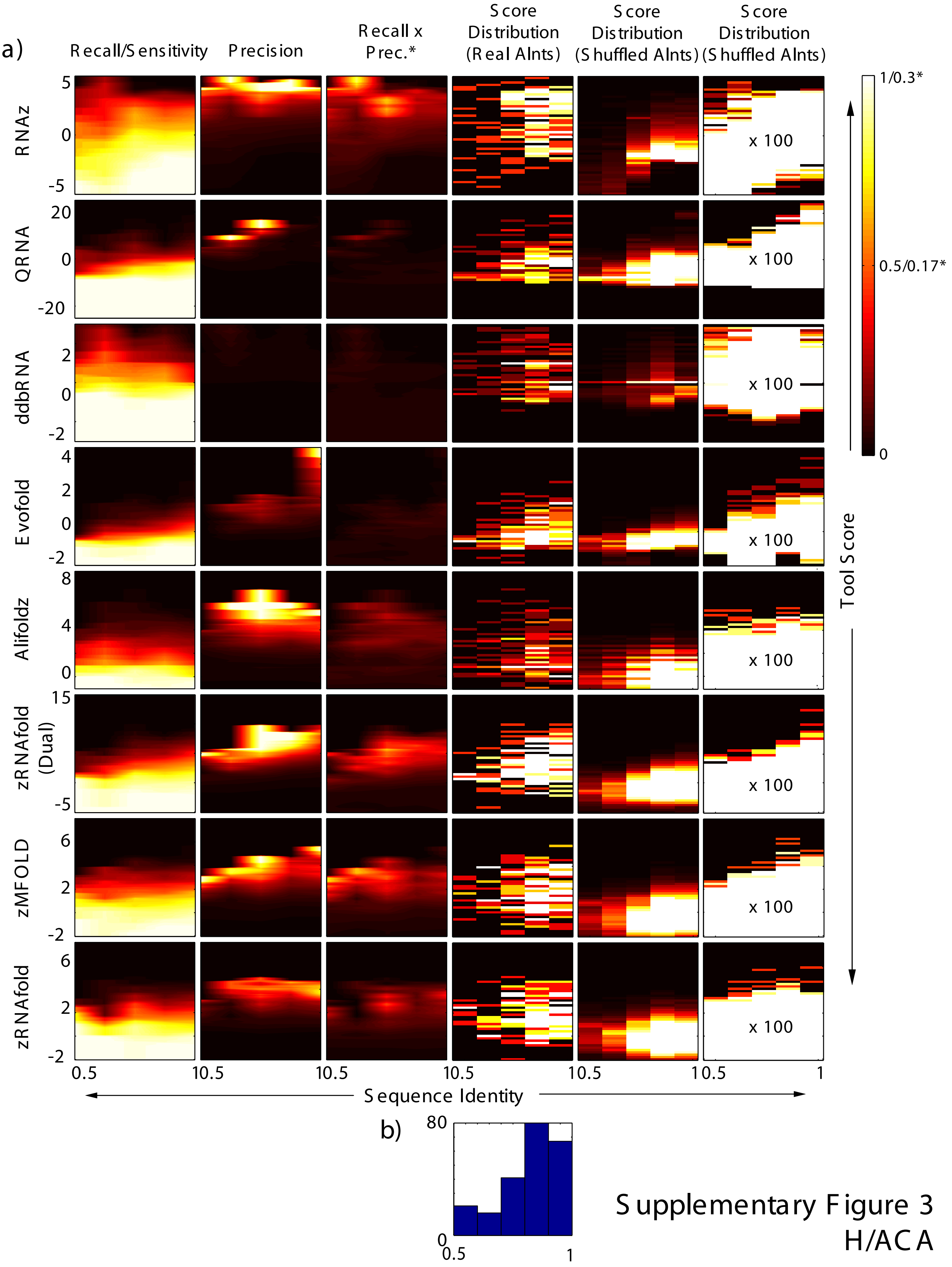

Supplementary Figure 3
H/ACA

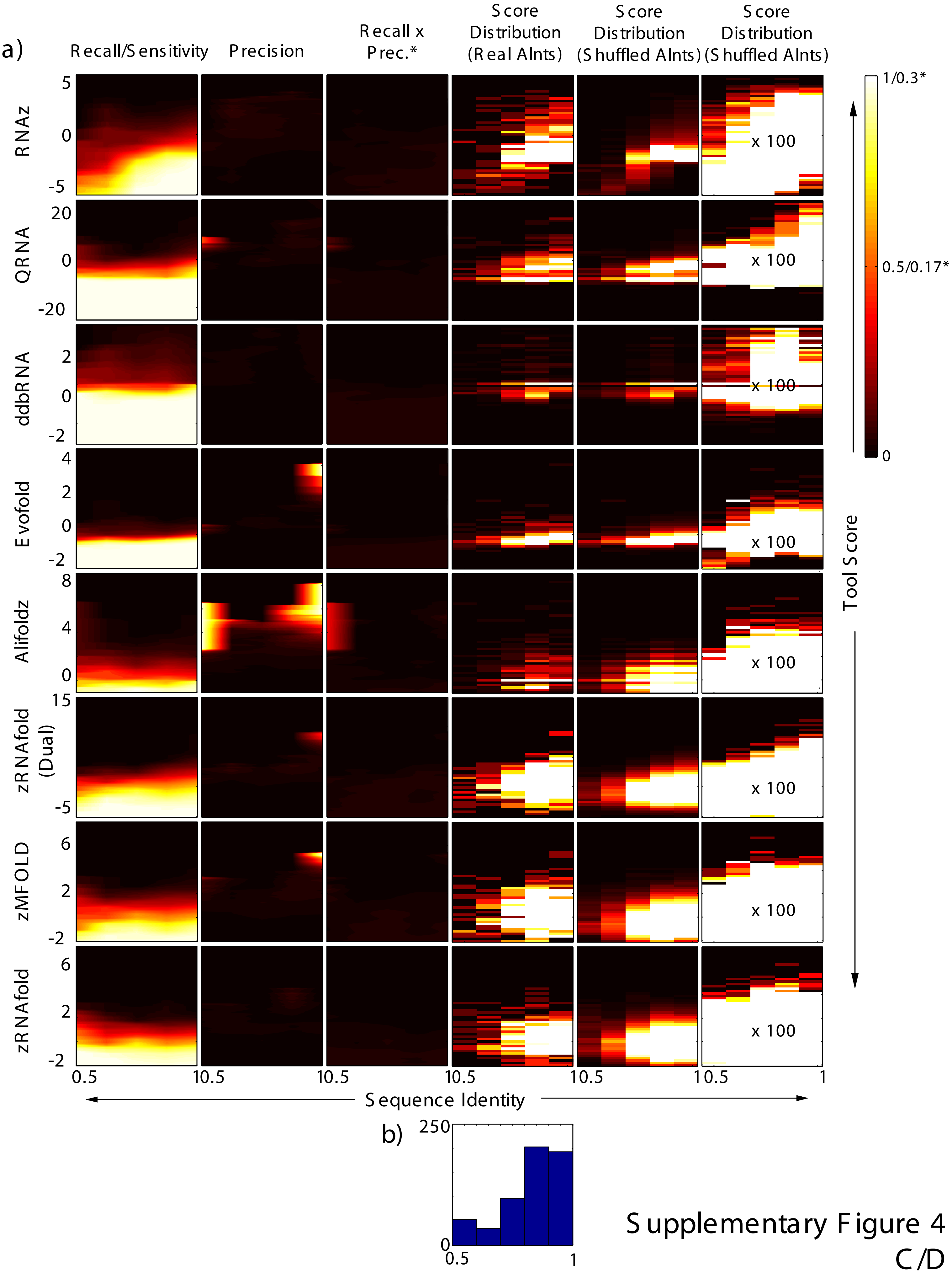

Supplementary Figure 4
C/D

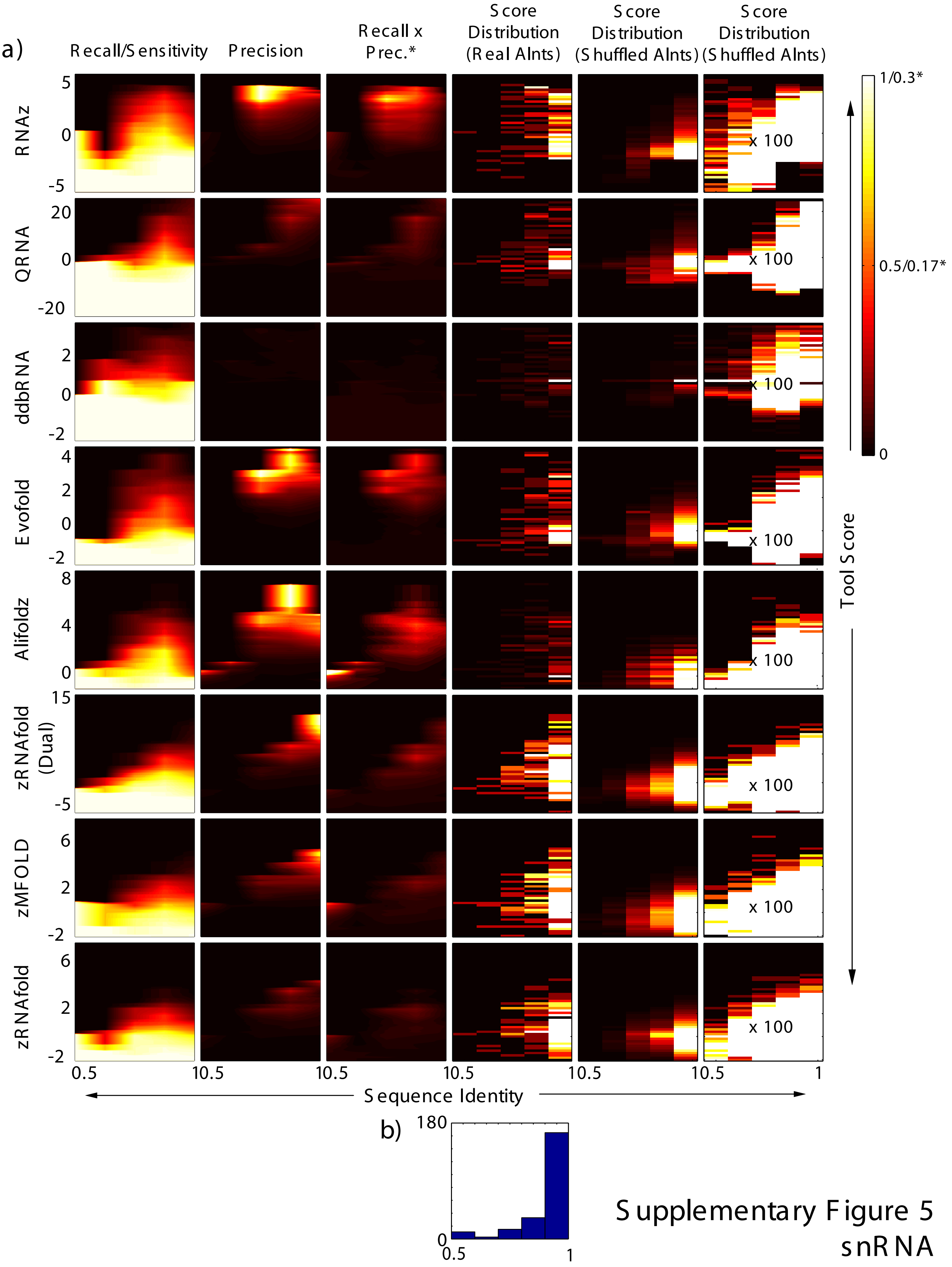

Supplementary Figure 5
snRNA

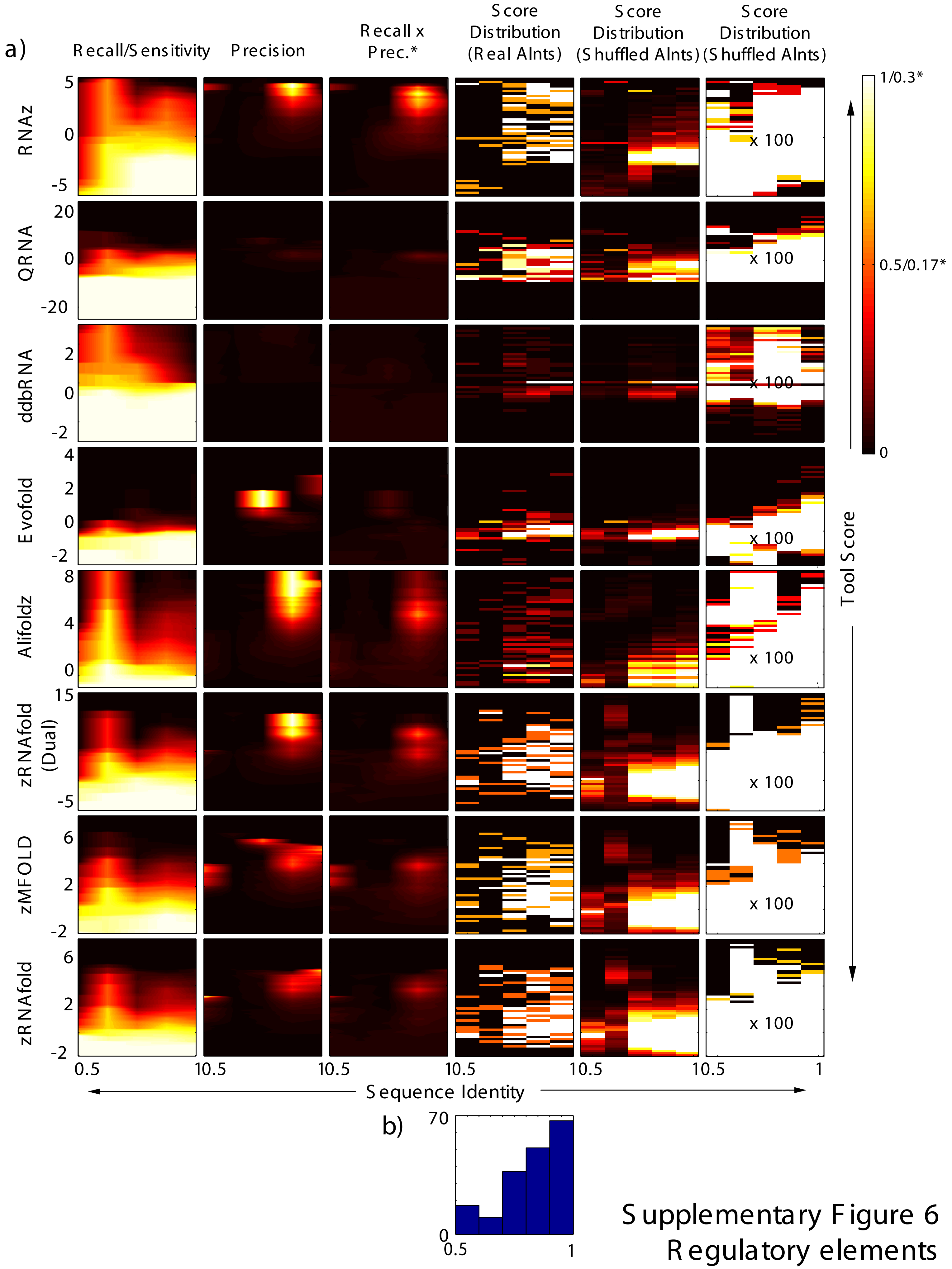

Supplementary Figure 6
Regulatory elements

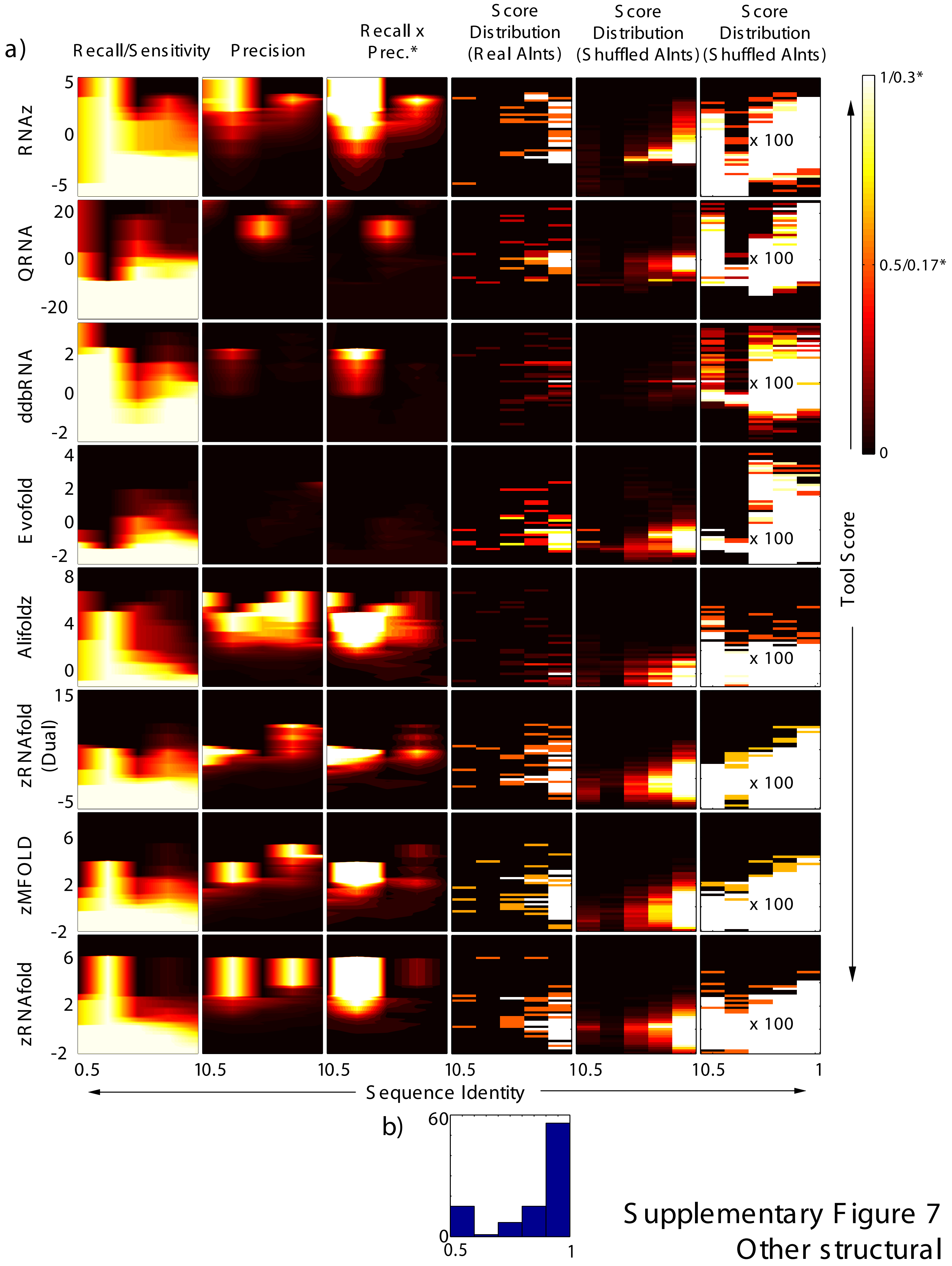

Supplementary Figure 7
Other structural

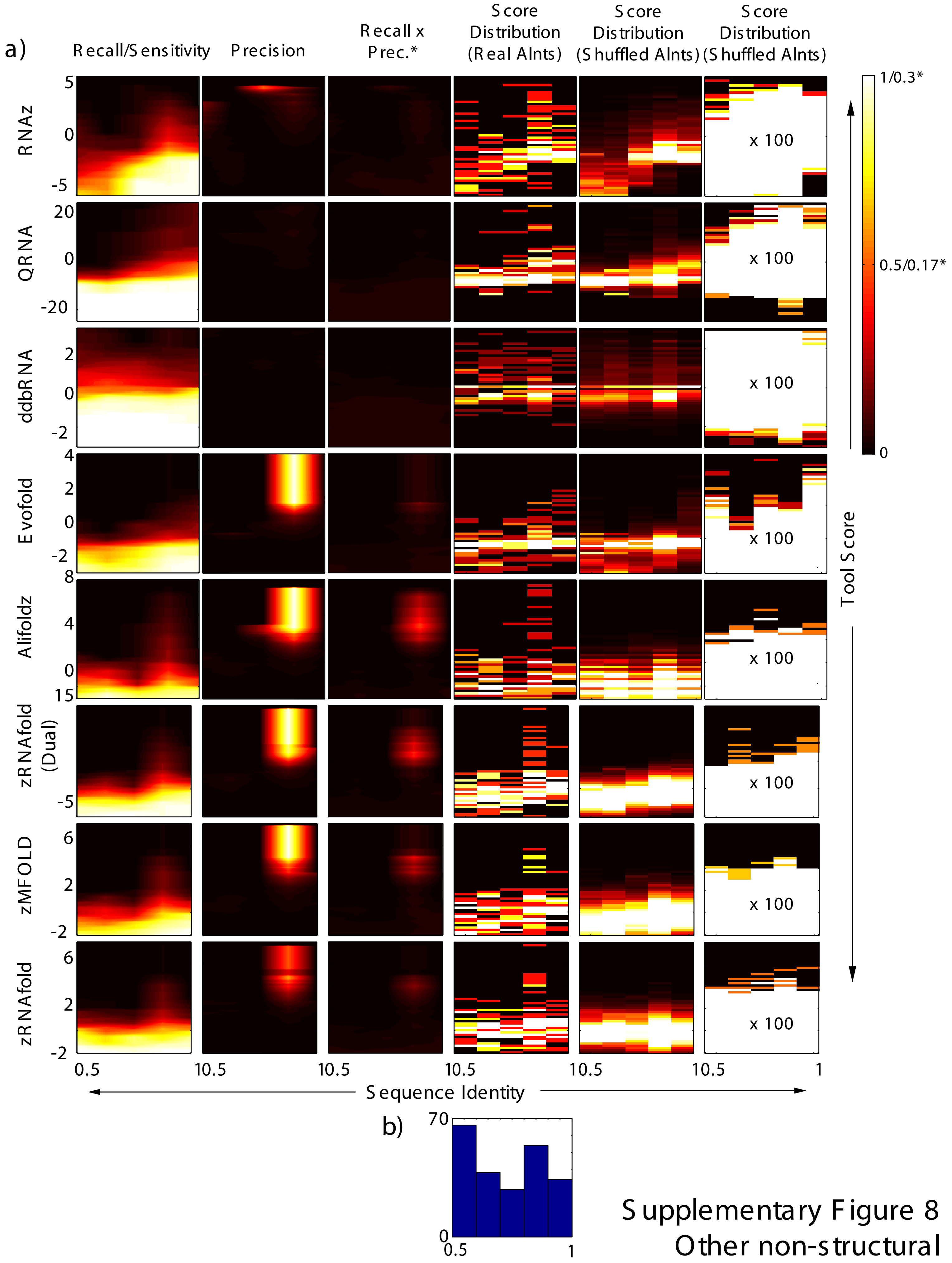

Supplementary Figure 8
Other non-structural
